# Supplementary material for: Control of complex networks requires both structure and dynamics
Source: Sci Rep. 2016 Apr 18;6:24456. doi: 10.1038/srep24456 (PMC4834509; doi:10.1038/srep24456)
Supplement: Supplementary Information [file srep24456-s1.pdf]

Supplemental Material for:

Control of complex networks requires both structure and dynamics

Alexander J. Gates and Luis M. Rocha

Contents

S1 Review of Network Structure based Control **5**
S1.1 Structural Controllability 5
S1.2 Minimal Dominated Sets 7

S2 Boolean Network Ensembles **7**
S2.1 Boolean Network Ensembles 7
S2.2 Determination of Dynamical Subset 8
S2.3 Extended Network Motif Analysis 8
S2.3.1 Feed-Forward motif 10
S2.3.2 Chain Motif 14
S2.3.3 Loop Motif 17
S2.3.4 Loop Motif with self-Interactions 20
S2.3.5 Fan Motif 23
S2.3.6 CoRegulated Motif 26
S2.3.7 CoRegulating Motif 29
S2.3.8 BiParallel Motif 32
S2.3.9 BiFan Motif 35
S2.3.10 Dominated Loop Motif 38

S3 Biological Networks **41**
S3.1 *Drosophila melanogaster* Segment Polarity Boolean Network 41
S3.2 *Saccharomyces cerevisiae* Cell Cycle Boolean Network 43
S3.3 *Arabidopsis thaliana* Floral Structure Boolean Network 46

S4 Canalization and Boolean Network Control **47**
S4.1 Canalization in *Saccharomyces cerevisiae* 50
S4.2 Canalization in Network Motifs 51

List of Figures

S1 All Network Motif Structural Graphs 9
S2 FeedForward Motif Control Profile 12
S3 FeedForward Motif Control Statistics 13
S4 Chain Motif Control Profile 15
S5 Chain Motif Control Statistics 16
S6 Loop Motif Control Profile 18
S7 Loop Motif Control Statistics 19
S8 Loop Motif with self-interactions Control Profile 21
S9 Loop Motif with self-interactions Control Statistics 22
S10 Fan Motif Control Profile 24
S11 Fan Motif Control Statistics 25
S12 CoRegulated Motif Control Profile 27
S13 CoRegulated Motif Control Statistics 28
S14 CoRegulating Motif Control Profile 30
S15 CoRegulating Motif Control Statistics 31
S16 BiParallel Motif Control Profile 33
S17 BiParallel Motif Control Statistics 34
S18 BiFan Motif Control Profile 36
S19 BiFan Motif Control Statistics 37
S20 Dominated Motif Control Profile 39
S21 Dominated Loop Motif Control Statistics 40
S22 *Drosophila melanogaster* Structural Graph 43
S23 *Saccharomyces cerevisiae* Structural Graph 44
S24 *Arabidopsis thaliana* Structural Graph 48
S25 *Arabidopsis thaliana* Control Analysis 49
S26 Canalization in the CCN Best-case 53
S27 Canalization in the CCN Worst-case 54
S28 Pure Loop Motif with self-interactions Control Profile 56
S29 Pure Loop Motif with self-interactions Control Statistics 57
S30 Pure CoRegulated Motif Control Profile 58
S31 Pure CoRegulated Motif Control Statistics 59
S32 Pure BiFan Motif Control Profile 60
S33 Pure BiFan Motif Control Statistics 61
S34 Pure Dominated Loop Motif Control Profile 62
S35 Pure Dominated Motif Control Statistics 63
S36 Canalization and PRES 64

List of Tables

S1 *Drosophila melanogaster* Logical Rules 42
S2 *Drosophila melanogaster* MDS Sets 44
S3 *Arabidopsis thaliana* MDS Sets 47
S4 Canalization and Boolean Network Control CCN 52

S1 Review of Network Structure based Control

S1.1 Structural Controllability

Mathematical control theory defines a time-invariant system as fully ***controllable*** if the system can pass from any configuration to any other configuration in finite time. In general, necessary and sufficient conditions for controllability do not exist for non-linear dynamical systems, such as those used to model bio-chemical dynamics. Instead, linear approximations of the original system are studied.

Consider the states of N variables given by the configuration vector $\boldsymbol{X}(t) = (x_1(t), \ldots, x_N(t))$. The network structure is expressed by a directed graph with the $N \times N$ connectivity matrix $\boldsymbol{A}$ and specific connection weights a_{ji} characterizing the directed interaction between variables n_i and n_j. For the purposes of control, M input variables $\boldsymbol{u}(t) = (u_1(t), \ldots, u_M(t))$ are added to the system with additional edges given by the $N \times M$ input connectivity matrix $\boldsymbol{B}$. The linear dynamics of the system are then given by the system of differential equations:

$$\frac{d\boldsymbol{X}(t)}{dt} = \boldsymbol{A}\boldsymbol{X}(t) + \boldsymbol{B}\boldsymbol{u}(t) \tag{1}$$

The above linear system, simply denoted by the matrix pair $(\boldsymbol{A}, \boldsymbol{B})$, is controllable if and only if the $N \times NM$ controllability matrix $\boldsymbol{C} = (\boldsymbol{B}, \boldsymbol{AB}, \boldsymbol{A}^2\boldsymbol{B}, \ldots, \boldsymbol{A}^{N-1}\boldsymbol{B})$ satisfies Kalman's controllability rank condition [1]. Kalman's controllability rank condition equates to requiring the controllability matrix to have full rank: rank $\boldsymbol{C} = N$. A case of little interest occurs when $M = N$; there are as many input signals as nodes so each node can be independently manipulated to the desired final configuration. Instead, we would like to find the minimum number of input variables M needed to control the network.

The problem appears more complicated when the exact values of the connection weights a_{ij} are difficult to measure or unknown altogether. Instead, the edge and input graphs are reduced to structured matrices $\boldsymbol{A}_{\text{struct}}$, $\boldsymbol{B}_{\text{struct}}$ which merely denote the presence or absence of an interaction but ignore its magnitude. This is done by replacing the non-zero entries of $\boldsymbol{A}, \boldsymbol{B}$ with arbitrary weights a_{ij} and b_{ij} while retaining all of the zero entries [2].

Lin [3] defined a network to be ***structurally controllable*** if the linear system defined by the pair of structural matrices $(\boldsymbol{A}_{\text{struct}}, \boldsymbol{B}_{\text{struct}})$ is controllable. Structurally controllable matrices are dense in the space of real-valued matrices[3]; a convenient property for working with real-world networks since it implies results are tolerant of experimental error. Lin [3] and Shields & Pearson [4] further showed there was a simple graph theoretic criteria for structural controllability: the combined graph describing the network and inputs must be spanned by cacti. Cacti are composed of two graph theoretic objects: stems (simple paths) and buds (elementary cycles with an additional edge directed towards a vertex of the cycle).

A graph is spanned by cacti if every variable is contained in either a stem or a bud. This has the interpretation of every variable being accessible, or on a directed path from an input variable[5]. The problem of finding spanning cacti on a directed graph can be mapped to a dual problem involving maximum matchings on a graph [6, 7]. In this

dual problem the directed graph is represented as a bipartite, undirected graph with two groups of nodes — one group denotes the 'in' connections while the other denotes the 'out' connections from the original graph. The covering of the original graph by cacti can then be determined via a maximum matching algorithm for bipartite graphs (e.g. the Hopcroft-Karp algorithm [8]). Nodes representing 'in'-directed edges which are not matched by the algorithm must be controlled via an input. These variables are called ***driver variables*** or ***driver nodes***. The resulting set of driver variables is not unique, but depends on the maximum matching found. However, since the matching is maximum, the cardinality of the set is unique and gives the minimum number of inputs required to make the system $(\boldsymbol{A}_{\text{struct}}, \boldsymbol{B}_{\text{struct}})$ completely controllable. The novelty of the method developed by Liu et al. [7] is the interpretation of the graph matching problems as an energy function for statistical mechanics. This allows for the calculation of the average number of driver nodes needed for structural controllability for an ensemble of statically similar networks. It is important to note that while the number M of input nodes $u_m(t)$ is equal to the number of driver variables, the latter are not necessarily the only nodes which must be manipulated by the former. Additional interventions from the input nodes, that were not captured by the driver node metric, might be required to fully control the cycles in the system.

A core assumption of structural controllability is that the structural matrices, which are a linear approximation for the original system, accurately characterize the system dynamics. Therefore, **all** interactions that define the dynamics must be specified by the structural matrix, including self-interactions. Using the structural matrix as the linear approximation for the system is akin to assuming each node is a linear integrator of signals transmitted over its edges (i.e. the nodal dynamics are specified by a weighted sum of the properties on nodes connected by incoming edges), an oversimplification of many important biological systems. These assumptions have important ramifications for interpreting the results of the structural controllability analysis, namely: they mischaracterize the effect of self-loops.

Structural controllability has been used to identify key banks in interbank lending networks [9], to inform the design of train service networks [10], and to relate circular network motifs to control in transcription regulatory networks [11]. SC has also been used to suggest that biological systems are harder to control and have appreciably different control profiles than social or technological systems [12, 5].

Implementation

This study followed Commault et al. [6]; the problem of finding spanning cacti on a directed graph was reinterpreted as the dual problem of finding a maximum matching on the bipartite representation. The graph maximum matching was then solved using the Hopcroft-Karp algorithm [8]. Code is available on the authors website.

S1.2 Minimal Dominated Sets

The Minimal Dominated Set method approaches the control problem from a different theoretical prospective. Under this interpretation, each node can influence adjacent nodes independently, but this signal cannot propagate any further. Driver variables are identified as members of the minimal set such that every other variable is separated by at most one interaction [13, 14]. The methodology has found success in solving problems many combinatorial optimization problems including transportation routing, scheduling, and facility location [14]. It has also been used to uncover potential anticancer drug combinations [15] and to highlight biologically significant variables in protein interaction networks [16]. It has also been used, for instance, to identify control variables in protein interaction networks [17, 16] and characterize how disease genes perturb the Human regulatory network [18].

Perhaps unsurprisingly, the maximum degree and degree distribution of the network has a large influence over the size of the minimum Dominated set [13]. The size of the MDS is also dependent on whether the network is directed or undirected; undirected network require relatively fewer controlled nodes [19].

Implementation

In general, the computation of a MDS is NP-hard. However, in both the directed and undirected cases, the computation of a MDS can be recast in an integer linear programming formulation and efficiently solved using optimized software [13, 19]. Enumeration of all possible MDSs for a given network can be recast as the enumeration of all minimal transversals in a hypergraph which can be solved in polynomial time only in some cases [20]. Instead, since all of the networks considered here are extremely small (< 20 nodes), a MDS was found and then the enumeration of all other sets of the same size was explored.

S2 Boolean Network Ensembles

S2.1 Boolean Network Ensembles

The set of all possible dynamical transition functions constrained by the fixed structural network $G = (X, E)$ can be considered independently for each node variable x_i. For each variable x_i of G with $k_i > 0$, there are 2^{k_i} possible input patterns and $|F_i| = 2^{2^{k_i}}$ possible transition functions, where $F_i \equiv \{f_i\}$. For instance, there are $|F| = 16$ possible logical functions of $k = 2$ inputs. The case of variables with no inputs ($k_i = 0$) requires additional consideration. In general Boolean network models of biochemical regulation, the value of a variable with no inputs is specified outside of the considered model; such variables are assumed to be in steady-state with respect to the temporal dynamics of the model. This is traditionally implemented in one of two ways, both of which are problematic for our treatment. The first uses a constant logic function to maintain a constant value. However,

this assumption predetermines the value of the constant; any change to the constant value would correspond to a model modification. Therefore, perturbations cannot be applied to such constants. The second uses the copy logic function to maintain the initial value of the variable. However, the copy function technically corresponds to a Boolean function with one input ($k_1 = 1$), leading some researchers to modify the network structure through the addition of a self-loop when formulating the BN model[21].

Here, we are concerned with the interplay between network structure and control, thus we only consider variables which can be manipulated given a fixed structure. Therefore, we maintain the steady-state assumption using the copy logic without modifying the network structure. If the network structure were to be modified in accordance with the second traditional implementation, the self-loops introduced into the structural network would affect the predictions of SC (but not MDS). Namely, SC is biased toward excluding variables with self-interactions from the set of potential driver variables. Finally, for a given structure G of $N = |X|$ variables, the full ensemble of BNs contains all possible combinations of variable logic functions $L = \prod_{i=1}^{N} |F_i|$.

S2.2 Determination of Dynamical Subset

As discussed in the main text, the full ensemble of Boolean Networks is partitioned into three subsets: the non-contingent subset (NC), the reduced effective structure subset (RES), and the full effective structure subset (FES). The NC subset contains any network with at least one variable whose transition function is non-contingent (either a tautology or a contradiction). The RES subset is composed of networks that are not already classified as NC and which have at least one variable whose transition function is fully canalizing. Fully canalizing functions are identified as those functions with at least one variable which does not appear in any of the prime implicants found using the the Quine-McCluskey algorithm [22]. The FES subset is composed of all remaining networks which are not classified as either NC or RES. This classification schema was motivated by identifying networks whose structural graph accurately captured the set of input-output relations used by the system dynamics. However, there are several other classification schemes which might be appropriate. For example, one could consider classifying networks once the control variables were specified, in which case the above criteria would only apply to transition functions of variables which were not being manipulated by the controller.

S2.3 Extended Network Motif Analysis

Here we explore the controllability of several three and four variable network motifs commonly found in molecular biology, neurobiology, ecology, electrical circuits [23, 24, 25], and the study of network controllability [5]. The motif structures are shown in Figure S1.

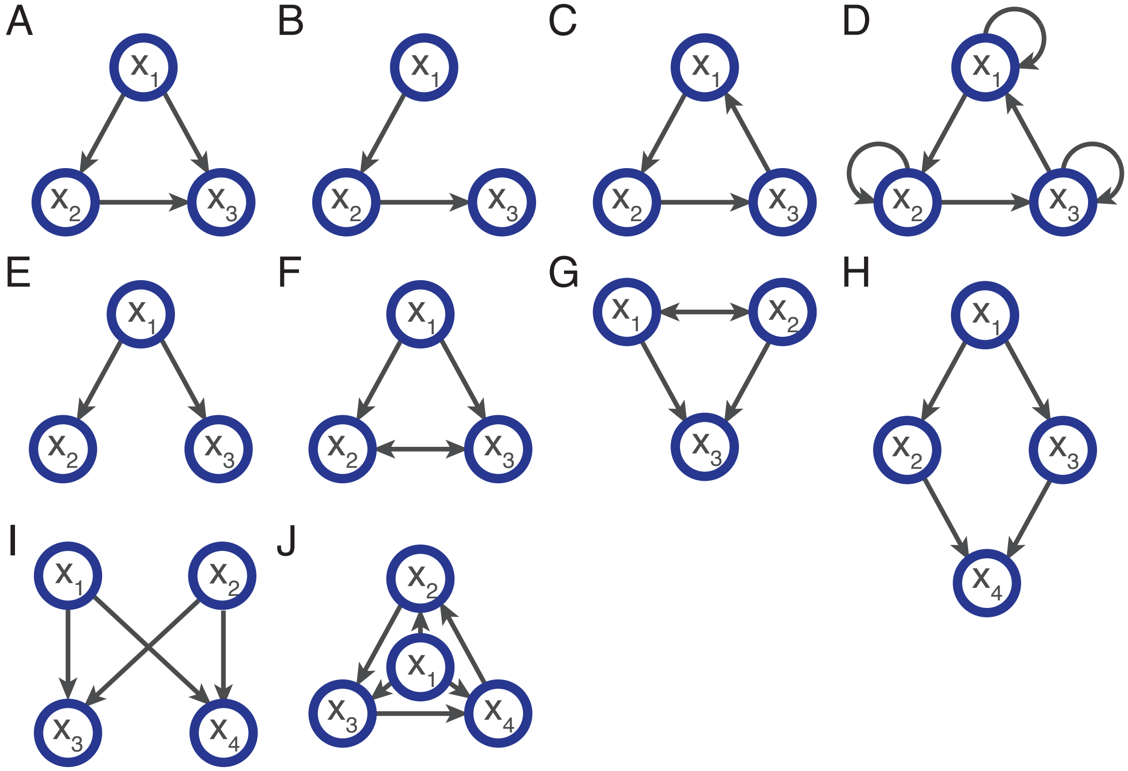

Figure S1: A) Feed-Forward motif, B) Chain motif, C) Loop motif, D) Loop motif with self-interactions, E) Fan motif, F) Co-regulated motif, G) Co-regulating motif, H) BiParallel motif, I) BiFan motif, and J) Dominated Loop motif.

S2.3.1 Feed-Forward motif

In the main text, we discussed the full ensemble of BN whose structure is given by the Feed-Forward network motif of $N = 3$ variables shown in Figure S1A. As discussed there, the full ensemble consists of 64 distinct BN of which 36 are NC, 8 have RES, and 20 have FES. Both structural control methodologies predict that variable x_1 is capable of fully controlling the network — i.e. facilitate transitions from any configuration to any other configuration. However, our analysis shown in Figure S2 reveals that only 8 networks from the ensemble (4 RES and 4 FES) can be fully controlled by this driver variable, identified by $\overline{R}_{\{x_1\}} = 1$. Indeed, $D \equiv \{x_1\}$ fails to fully control 56 out of 64 possible BNs with the same structure, since $\overline{R}_{\{x_1\}} < 1$ for most BNs. To guarantee full control for every possible contingent network, x_1 and either x_2 or x_3 need to be driven ($D \equiv \{x_1, x_2\}, \{x_1, x_3\}$). When the full ensemble is considered, all three variables need to be driven to render every possible network fully controllable.

The trends in the control profiles can be better understood through comparison of the population statistics shown in Figure S3. Here, we can also clearly see that the driver variable x_1 predicted by structural control is better than the other two variables at controlling the full ensemble; the average of $\overline{R}_{x_1}$ over all networks in the full ensemble is 0.65 compared to an ensemble averaged $\overline{R}_{x_2}$ of 0.36 or an ensemble averaged $\overline{R}_{x_3}$ of 0.29. If we consider the fraction of additional transitions induced by interventions to the different driver variable sets as measured by $\overline{C}_D$, we see that the average of $\overline{C}_{x_1}$ over all networks in the full ensemble is 0.51 compared to an ensemble averaged $\overline{C}_{x_2}$ of 0.21 or an ensemble averaged $\overline{C}_{x_3}$ of 0.14. Indeed, from the point of view of attractor control, the single-variable driver set $D \equiv \{x_1\}$ can control every network ($\overline{A}_D = 1.0$) in the full ensemble regardless of the dynamical subset. Neither of the other two variables, $D \equiv \{x_2\}, \{x_3\}$, can push the dynamics from one attractor to another in any BN in the ensemble ($\overline{A}_D = 0.0$).

The Feed-Forward network motif has been suggested as a sign-sensitive accelerator or delay in biological transcription networks [26]. In this role, the suggested logic for variable x_3 is either AND or OR transition functions. It is interesting to note that, contrary to the predictions of structural control, neither of these transition functions are associated with networks that can be fully controlled by a single driver variable.

Nonetheless, the driver variable x_1 predicted by structural control is clearly better than the other two variables at controlling the full ensemble; the average of $\overline{R}_{\{x_1\}}$ over all networks in the full ensemble is 0.65 compared to an ensemble averaged $\overline{R}_{\{x_2\}}$ of 0.36 or an ensemble averaged $\overline{R}_{\{x_3\}}$ of 0.29; similar results hold for $\overline{C}_D$ as seen in Figure S3. Indeed, from the point of view of attractor control, the single-variable driver set $D \equiv \{x_1\}$ can control every network in the full ensemble ($\overline{A}_D = 1.0$); while neither of the other two variables, $D \equiv \{x_2\}, \{x_3\}$, can push the dynamics from one attractor to another in any BN in the ensemble ($\overline{A}_D = 0.0$).

Regarding the partitions of the BN ensemble, we can see that when $D \equiv \{x_1\}$, controllability is on average higher for the FES subset than for the RES and non-contingent cases. In other words, when the effective structure of interactions is least distinct from the original interaction structure — when there are no fully canalizing or non-contingent

functions — structure is naturally better at predicting control. This becomes even more clear when we observe that the 4 fully controllable FES networks all contain an XOR transition function on variable x_3, a function that always requires both inputs to be resolved (no canalization at all). Similarly, the 4 fully controllable RES networks all treat the input from x_1 to x_3 as redundant, effectively reducing the structural graph to the chain motif shown in Figure S1B with contingent functions on all variables which is always fully controllable (see Figure S3). Thus, while structure is clearly insufficient to predict control of the dynamics that can unfold on this motif (even for a majority of FES networks), it does play a role.

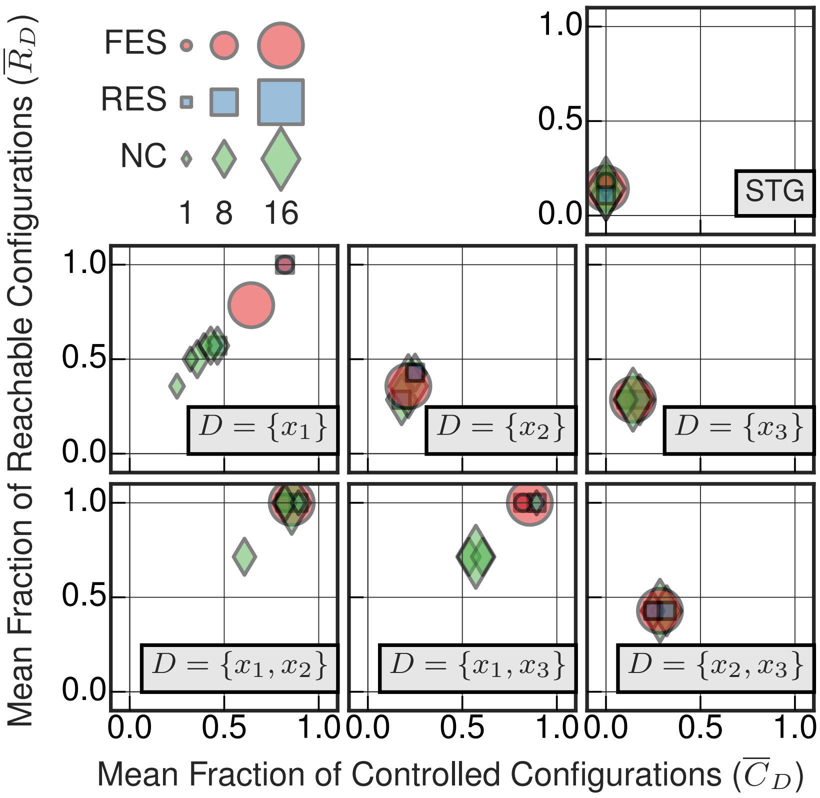

Figure S2: The average fraction of reachable configurations $\overline{R}$ and the average fraction of controllable configurations $\overline{C}$ for the full ensemble of 64 BN with structure given by the $N = 3$ variable Feed-Forward network motif shown in Figure S1A, as controlled by the driver variable sets $D \equiv \{x_1\}, \{x_2\}, \{x_3\}, \{x_1, x_2\}, \{x_1, x_3\}, \{x_2, x_3\}$. There are 20 networks in the full effective structure (FES) subset shown with red circles, 8 networks in the reduced effective structure (RES) subset shown with blue squares, and 36 networks in the NC subset shown with green diamonds; the area of the object corresponds to the number of networks. Both SC and MDS methods predict $D \equiv \{x_1\}$ is sufficient to fully control the system dynamics.

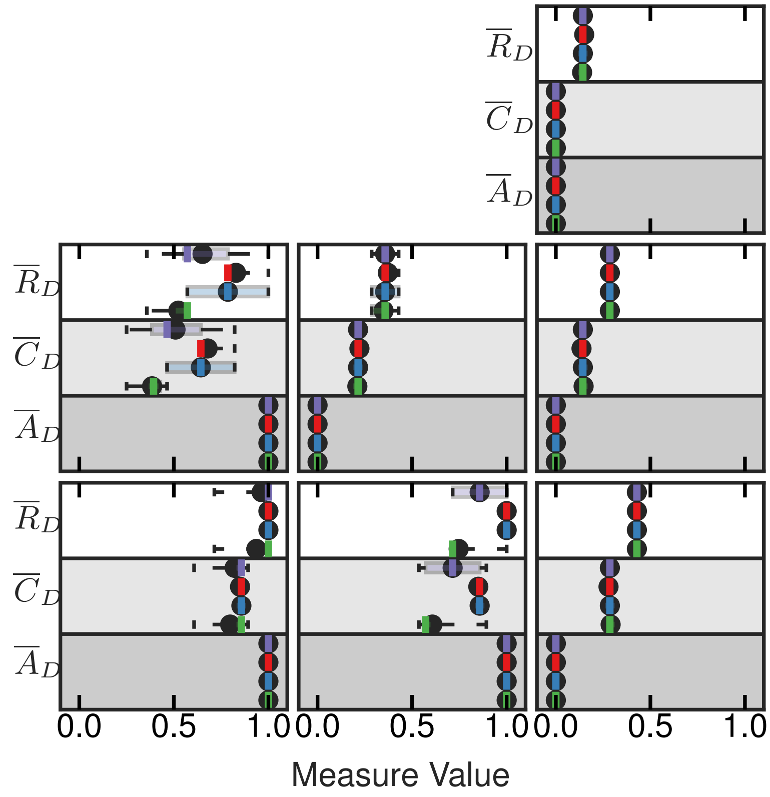

Figure S3: Boxplots summarizing the controllability measures for the Feed-Forward network motif. These population statistics are shown for the Full Ensemble (purple), FES (red), RES (blue), and NC (green) subsets. The mean values for each measure and subset are denoted by black dots, the median values for each measure and subset are shown by the appropriately colored solid line, the box illustrates the interquartile range, and the whiskers denote the minimum and maximum values. Subplots correspond to the same driver variable sets as shown in Figure S2.

S2.3.2 Chain Motif

Another network motif found in many real-world systems is the $N = 3$ variable Chain network motif [27], specified by the structural network shown in Figure S1B. For this network structure, there are 16 networks in the full ensemble of which 4 networks are in the FES subset, 0 networks are in the RES subset, and 12 networks are in the NC subset. The chain motif provides the first example where the two structural control methods (SC and MDS) offer different predictions: SC predicts $D \equiv \{x_1\}$ is sufficient to fully control the system dynamics, while MDS predicts two variables (either $D \equiv \{x_1, x_2\}, \{x_1, x_3\}$) are necessary. Analysis of the control profiles presented in Figure S4 shows that all of the FES networks are fully controllable ($\overline{R} = 1.0$) from interventions to the driver variable set $D \equiv \{x_1\}$ as predicted by SC. Interventions to this driver variable account for a large number of additional transitions in the CSTG as quantified by large values of $\overline{C} = 0.82$ for all networks in the FES subset. Further population statistics for each of the measures are summarized in Figure S5.

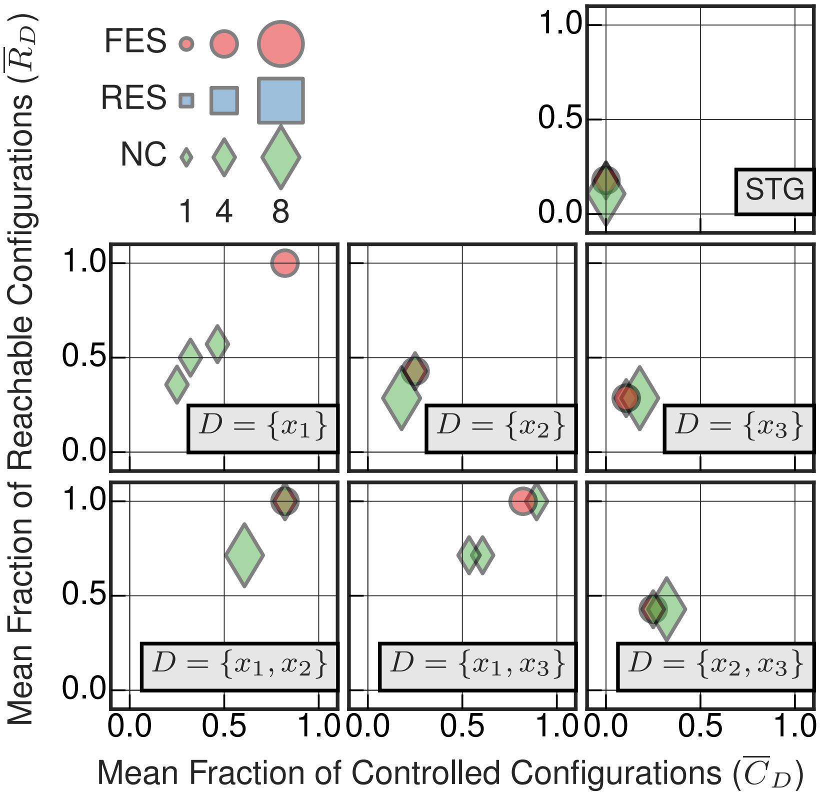

Figure S4: The average fraction of reachable configurations $\overline{R}$ and the average fraction of controllable configurations $\overline{C}$ for the full ensemble of 16 BN with structure given by the $N = 3$ variable Chain network motif shown in Figure S1B, as controlled by the driver variable sets $D \equiv \{x_1\}, \{x_2\}, \{x_3\}, \{x_1, x_2\}, \{x_1, x_3\}, \{x_2, x_3\}$. There are 4 networks in the FES subset shown in red circles, 0 networks in the RES subset, and 12 networks in the NC subset shown in green diamonds; the area of the object corresponds to the number of networks. SC predicts $D \equiv \{x_1\}$ is sufficient to fully control the system dynamics, while MDS predicts two variables (either $D \equiv \{x_1, x_2\}, \{x_1, x_3\}$) are necessary.

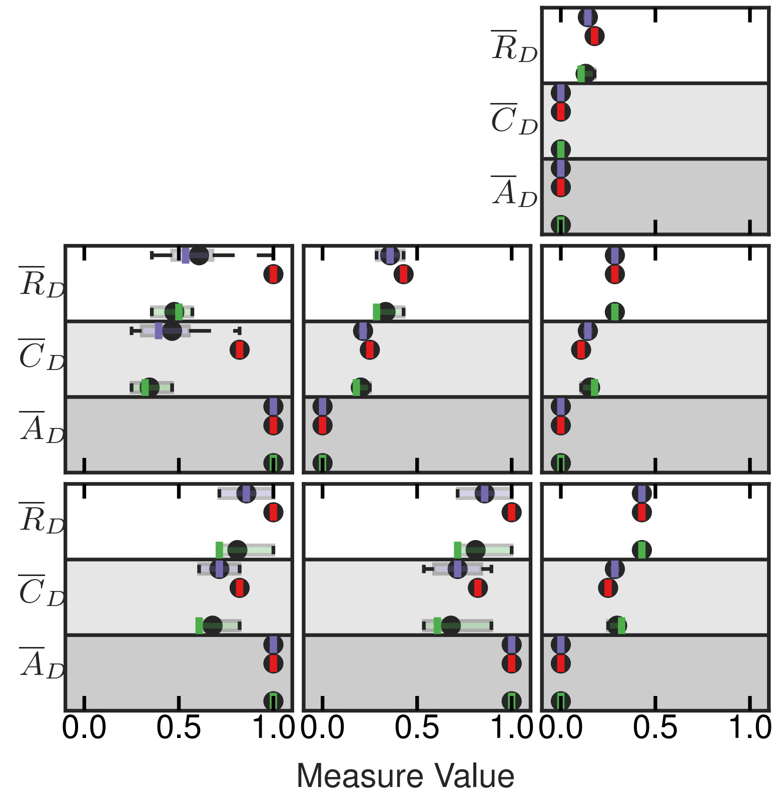

Figure S5: Boxplots summarizing the controllability measures for the Chain network motif. These population statistics are shown for the Full Ensemble (purple), FES (red), and NC (green) subsets. The mean values for each measure and subset are denoted by black dots, the median values for each measure and subset are shown by the appropriately colored solid line, the box illustrates the interquartile range, and the whiskers denote the minimum and maximum values. Subplots correspond to the same driver variable sets as shown in Figure S4.

S2.3.3 Loop Motif

The Loop motif is shown in Figure S1C. Its full ensemble also consists of 64 distinct BN of which 56 are in the NC subset, 0 are in the RES subset, and 8 are in the FES subset. The control profiles for the full ensemble is shown in Figure S6 when $D \equiv \{x_1\}, \{x_2\}, \{x_3\}$ and $D \equiv \{x_1, x_2\}, \{x_1, x_3\}, \{x_2, x_3\}$. The population statistics for each of the measures are summarized in Figure S7. Again, the SC analysis predicts that any single variable is capable of fully controlling the network, while the MDS analysis predicts two variables are needed to fully control the network. Our controllability analysis shows that a single driver variable ($D \equiv \{x_1\}$) or any pair of variables ($D \equiv \{x_1, x_2\}$) fail to fully control a large majority of the possible BN; to guarantee full control for every network in the dynamical ensemble constrained by the Loop network structure, all three variables need to be driven. However, it is interesting to note that, as in the case of the Chain motif, the FES subset of network dynamics are fully controllable from just one driver variable. This subset is split into two groups by the mean fraction of controlled configurations (depending on the parity of the Boolean functions [28]).

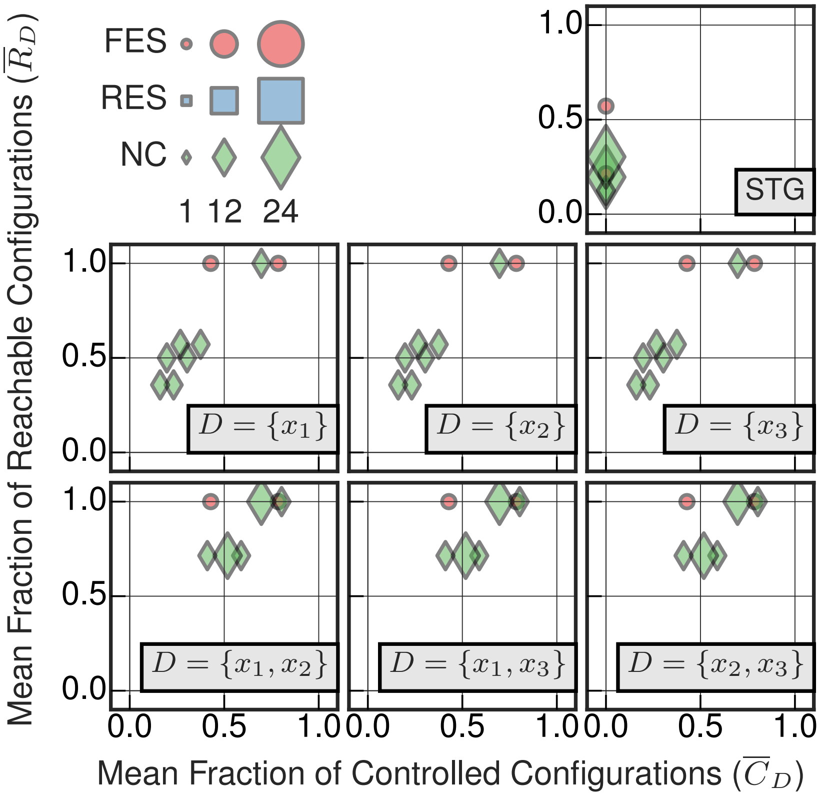

Figure S6: The average fraction of reachable configurations $\overline{R}$ and the average fraction of controllable configurations $\overline{C}$ for the full ensemble of 64 BN with structure given by the $N = 3$ variable Loop network motif shown in Figure S1C, as controlled by the driver variable sets $D \equiv \{x_1\}, \{x_2\}, \{x_3\}, \{x_1, x_2\}, \{x_1, x_3\}, \{x_2, x_3\}$. There are 8 networks in the FES subset shown in red circles, 0 networks in the RES subset, and 56 networks in the NC subset shown in green diamonds; the area of the object corresponds to the number of networks. SC predicts any singleton set ($D \equiv \{x_1\}, \{x_2\}, \{x_3\}$) is sufficient to fully control the system dynamics, while MDS predicts any two variables ($D \equiv \{x_1, x_2\}, \{x_1, x_3\}, \{x_2, x_3\}$) are necessary.

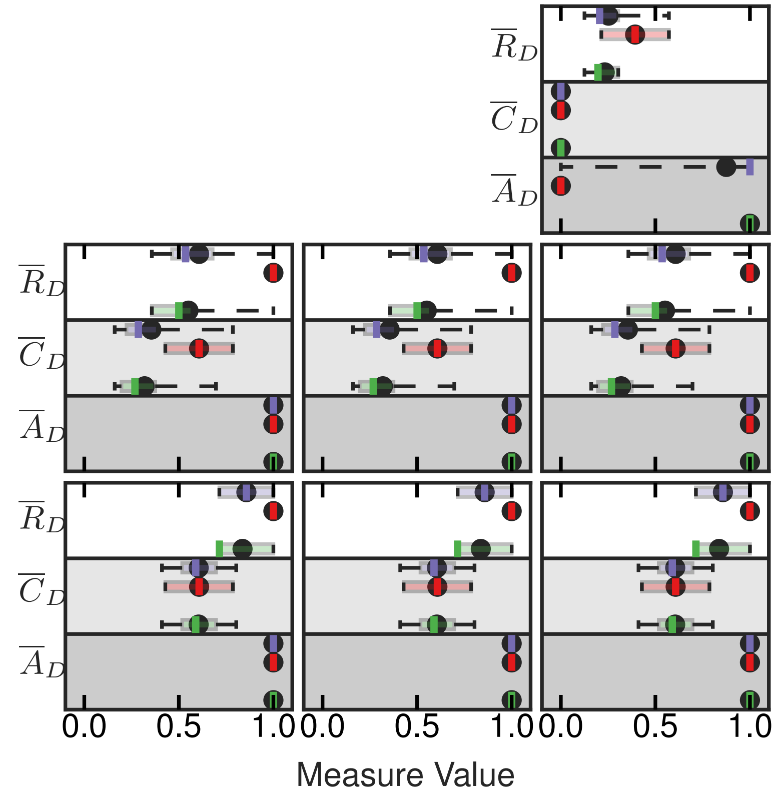

Figure S7: Boxplots summarizing the controllability measures for the Loop network motif. These population statistics are shown for the Full Ensemble (purple), FES (red), and NC (green) subsets. The mean values for each measure and subset are denoted by black dots, the median values for each measure and subset are shown by the appropriately colored solid line, the box illustrates the interquartile range, and the whiskers denote the minimum and maximum values. Subplots correspond to the same driver variable sets as shown in Figure S6.

S2.3.4 Loop Motif with self-Interactions

The full ensemble of BN whose structure is given by the $N = 3$ variable Loop network motif with self-interactions (shown in Figure S1D) was discussed in the main text. Here, we explicitly show the control profiles and population statistics for all driver variable sets in Figures S8 and S9.

The control portraits in Figure S8 again demonstrate that structure fails to characterize network control. There is a wide variation of both $\overline{R}_D$ and $\overline{C}_D$ values for all possible networks in the ensemble. In this case, SC predicts that any single variable is sufficient for full controllability, while MDS requires any two variables to achieve the same. Yet controllability varies greatly for both cases, depending on the particular transition functions of each BN in the ensemble. For 77% (3168 of 4096) of the BN in the ensemble a single variable is not capable of fully controlling dynamics; even two-variable driver sets fail to control 44% (1792 of 4096) of the BN. We also observe that while the RES subset contains networks with less controllability (measured by $\overline{R}_D$ or $\overline{C}_D$), both the RES and FES subsets display large variation (Figure 4A). In particular, the FES subset — most coherent with structural control — again contains many networks which are not fully controllable as predicted by SC and MDS.

Similar results hold for the mean fraction of reachable attractors ($\overline{A}_D$) shown in Figure 4B (middle, right). For 36% (1456 of 4096) of the BNs in the ensemble, a single variable is not capable of fully controlling the system between attractors; even two-variable driver sets fail to control 20% (808 of 4096) of the BNs, regardless of the dynamical subset.

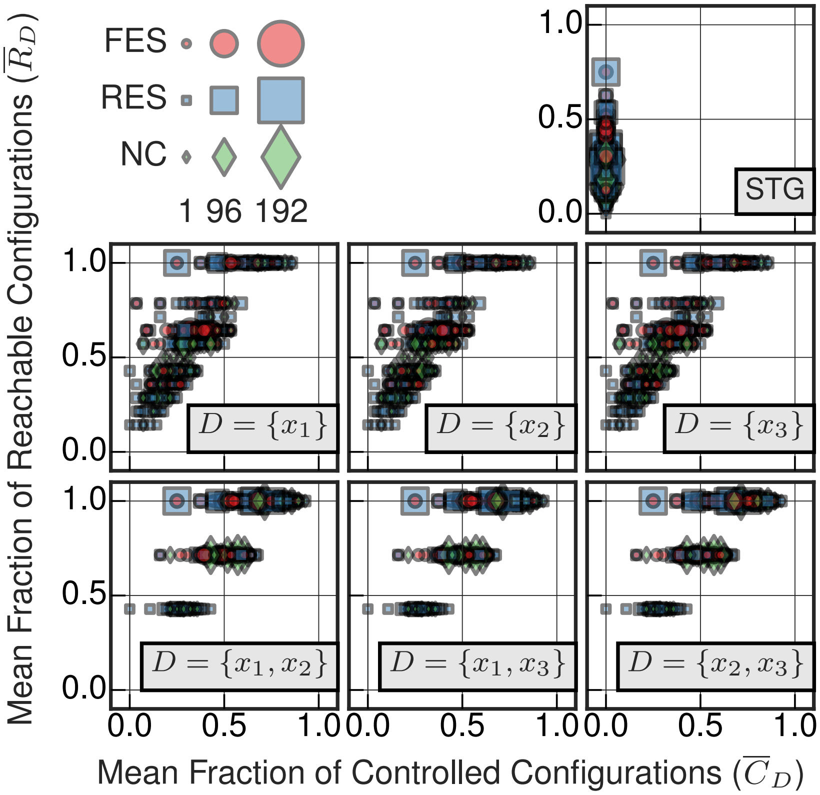

Figure S8: The average fraction of reachable configurations $\overline{R}$ and the average fraction of controllable configurations $\overline{C}$ for the full ensemble of 4096 BN with structure given by the $N = 3$ variable Loop network motif with self interactions, shown in Figure S1D, as controlled by the driver variable sets $D \equiv \{x_1\}, \{x_2\}, \{x_3\}, \{x_1, x_2\}, \{x_1, x_3\}, \{x_2, x_3\}$. There are 1000 networks in the FES subset shown in red circles, 1744 networks in the RES subset shown in blue squares, and 1352 networks in the NC subset shown in green diamonds; the area of the object corresponds to the number of networks. SC predicts any singleton set ($D \equiv \{x_1\}, \{x_2\}, \{x_3\}$) is sufficient to fully control the system dynamics, while MDS predicts any two variables ($D \equiv \{x_1, x_2\}, \{x_1, x_3\}, \{x_2, x_3\}$) are necessary.

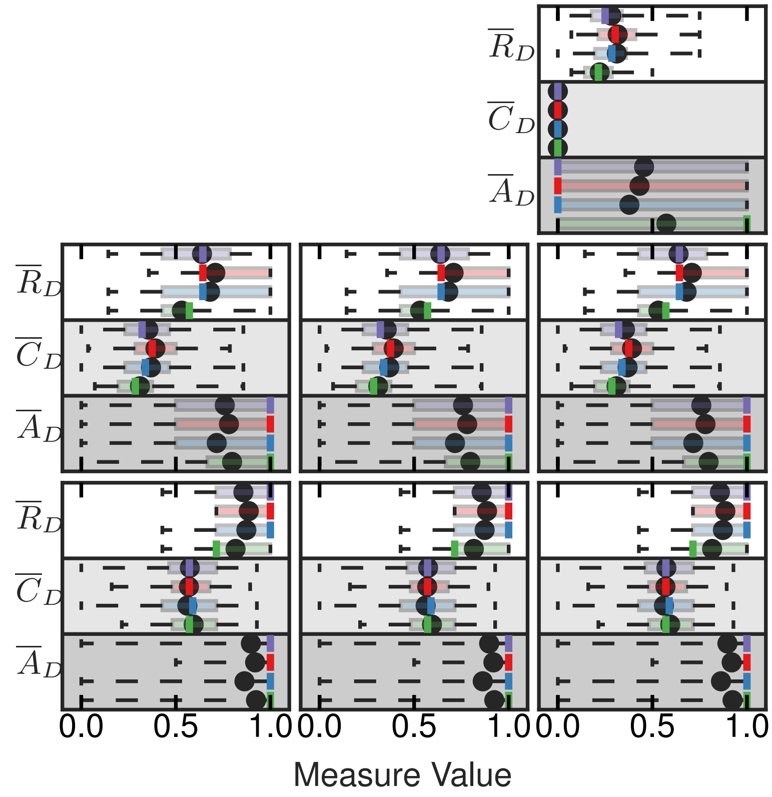

Figure S9: Boxplots summarizing the controllability measures for the Loop network motif with self interactions. These population statistics are shown for the Full Ensemble (purple), FES (red), RES (blue) and NC (green) subsets. The mean values for each measure and subset are denoted by black dots, the median values for each measure and subset are shown by the appropriately colored solid line, the box illustrates the interquartile range, and the whiskers denote the minimum and maximum values. Subplots correspond to the same driver variable sets as shown in Figure S8.

S2.3.5 Fan Motif

The full ensemble of BN whose structure is given by the $N = 3$ variable Fan network motif (specified in Figure S1E) consists of 4 BNs with FES and 12 BNs with NC. The Fan motif is the smallest example from the family of motifs known as single-input modules [25], commonly found in gene regulatory networks and associated with coordinated expression of genes with a shared expression. From a structural controllability viewpoint, the Fan motif is the smallest example of a network with a dilation [5]. This dilation motivates SC to predict that two variables are needed for fully controlling the network ($D \equiv \{x_1, x_2\}, \{x_1, x_3\}$), while MDS predicts only one variable is needed to fully control the network ($D \equiv \{x_1\}$). The control profiles in Figure S10 clearly demonstrate that controlling any one variable in the network fails to fully control any of the networks in the full ensemble. However, controlling variable $D \equiv \{x_1\}$ is sufficient to control every BN between its two attractors, as shown by the population statistics in Figure S11. Interventions to the two variable sets ($D \equiv \{x_1, x_2\}, \{x_1, x_3\}$) renders all of the BNs in the FES subset fully controlled, while interventions to all three variables are needed to fully control all of the BN in the full ensemble.

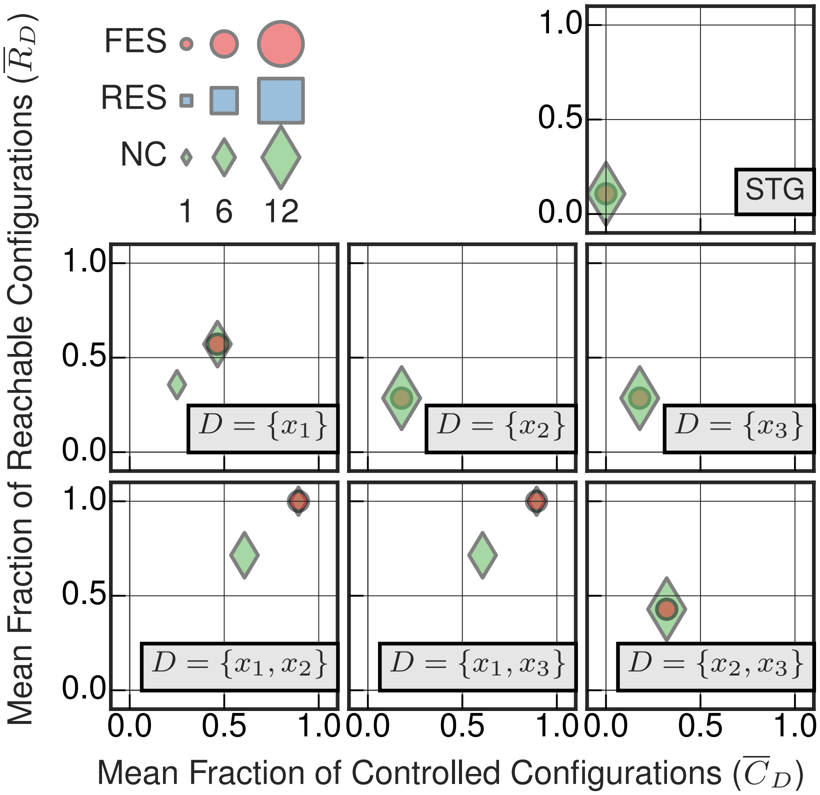

Figure S10: The average fraction of reachable configurations $\overline{R}$ and the average fraction of controllable configurations $\overline{C}$ for the full ensemble of 16 BN with structure given by the $N = 3$ variable Fan network motif shown in Figure S1E, as controlled by the driver variable sets $D \equiv \{x_1\}, \{x_2\}, \{x_3\}, \{x_1, x_2\}, \{x_1, x_3\}, \{x_2, x_3\}$. There are 4 networks in the FES subset shown in red circles, 0 networks in the RES subset, and 12 networks in the NC subset shown in green diamonds; the area of the object corresponds to the number of networks. The SC analysis predicts $D \equiv \{x_1, x_2\}, \{x_1, x_3\}$ while the MDS analysis predicts $D \equiv \{x_1\}$ is sufficient to fully control the system dynamics.

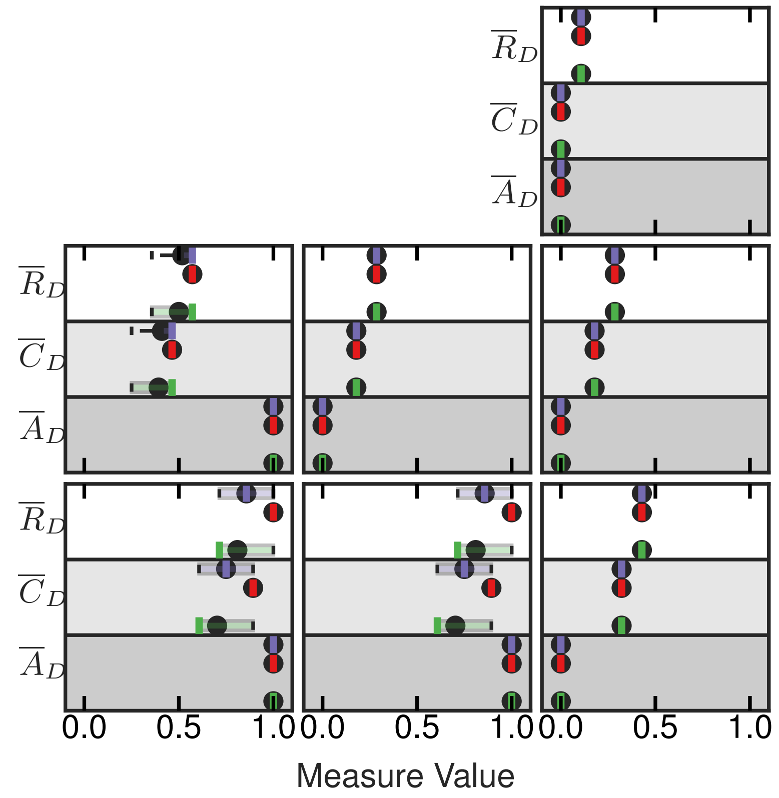

Figure S11: Boxplots summarizing the controllability measures for the Loop network motif with self interactions. These population statistics are shown for the Full Ensemble (purple), FES (red), and NC (green) subsets. The mean values for each measure and subset are denoted by black dots, the median values for each measure and subset are shown by the appropriately colored solid line, the box illustrates the interquartile range, and the whiskers denote the minimum and maximum values. Subplots correspond to the same driver variable sets as shown in Figure S10.

S2.3.6 CoRegulated Motif

The coregulated network motif, shown in Figure S1F, is statistically overrepresented in several composite networks of gene regulation and protein-protein interaction [24]. There are 256 BN in the full ensemble of which 100 networks are in the FES subset, 96 networks are in the RES subset, and 60 networks are in the NC subset. Based solely on the structural network, both SC and MDS predict interventions on a single variable ($D \equiv \{x_1\}$) are sufficient to fully control the network. However, this driver variable set is only capable of fully controlling 76 of the BN in the full ensemble (34 FES and 42 RES) as seen in the control profiles in Figure S12.

The analysis of attractor controllability for this network ensemble (shown in Figure S13) shows that a necessary condition for full attractor control is having $\{x_1\}$ as a subset of the driver variable set. However, this is not a sufficient condition for attractor controllability. To understand why, we must consider that there are 136 networks with 2 attractors, 116 networks with 4 attractors, and 4 networks with 6 attractors.

This network motif also provides an interesting example of a control variable whose manipulation does not facilitate any additional transitions in the CSTG, thus the average fraction of controllable configurations $\overline{C} = 0$ even though $\overline{R} > 0$. Here, we see this occurring for a small subset of 4 networks when $D \equiv \{x_2\}, \{x_3\}$.

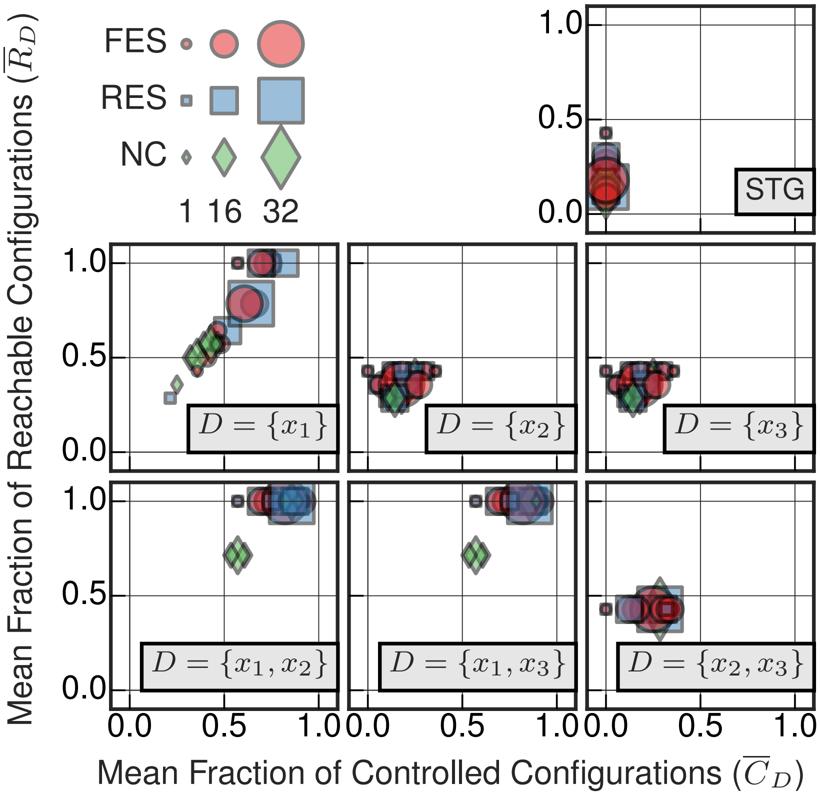

Figure S12: The average fraction of reachable configurations $\overline{R}$ and the average fraction of controllable configurations $\overline{C}$ for the full ensemble of 256 BN with structure given by the $N = 3$ variable CoRegulated network motif shown in Figure S1F, as controlled by the driver variable sets $D \equiv \{x_1\}, \{x_2\}, \{x_3\}, \{x_1, x_2\}, \{x_1, x_3\}, \{x_2, x_3\}$. There are 100 networks in the FES subset shown in red circles, 96 networks in the RES subset shown in blue squares, and 60 networks in the NC subset shown in green diamonds; the area of the object corresponds to the number of networks. Both SC and MDS methods predict $D \equiv \{x_1\}$ is sufficient to fully control the system dynamics.

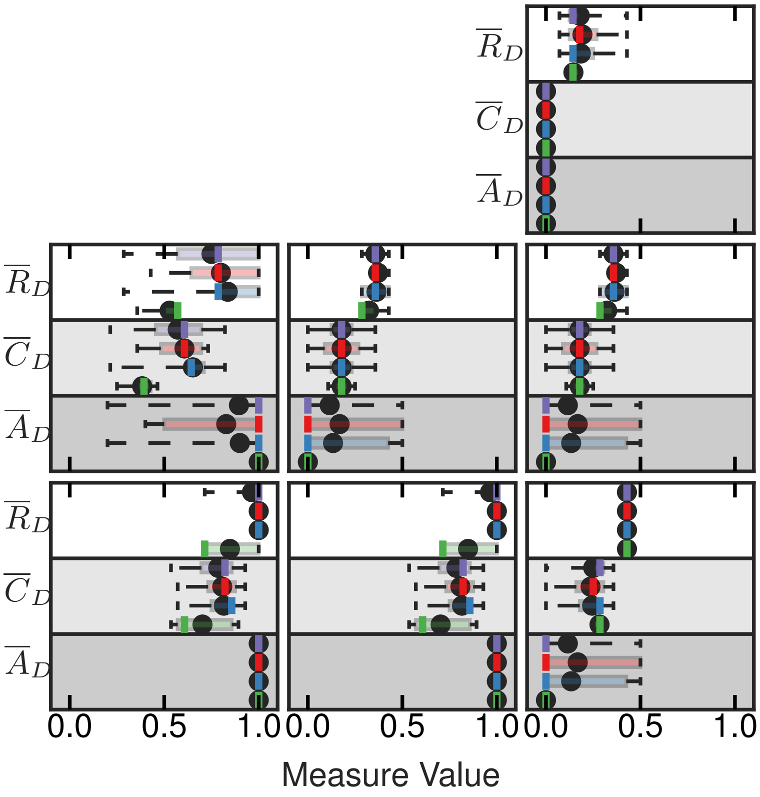

Figure S13: Boxplots summarizing the controllability measures for the CoRegulated network motif. These population statistics are shown for the Full Ensemble (purple), FES (red), and NC (green) subsets. The mean values for each measure and subset are denoted by black dots, the median values for each measure and subset are shown by the appropriately colored solid line, the box illustrates the interquartile range, and the whiskers denote the minimum and maximum values. Subplots correspond to the same driver variable sets as shown in Figure S12.

S2.3.7 CoRegulating Motif

The coregulating network motif, shown in Figure S1G, has also been identified in composite networks of gene regulation and protein-protein interaction [24]. There are 256 BN in the full ensemble of which 40 networks are in the FES subset, 16 networks are in the RES subset, and 200 networks are in the NC subset. Based solely on the structural network, both SC and MDS predict interventions on a single variable ($D \equiv \{x_1\}, \{x_2\}$) are sufficient to fully control the network. However, both of these driver variable sets are only capable of fully controlling 32 of the BN in the full ensemble (8 FES, 8 RES, and 16 NC) as seen in the control profiles in Figure S14. The attractor control analysis, presented in Figure S15, should only be considered in light of the fact that the majority of networks in this ensemble have only 1 attractor (hence $\overline{A} = 1$ by default). The remaining 12 networks have 3 attractors, which can fully controlled by interventions to the structurally predicted driver variable sets.

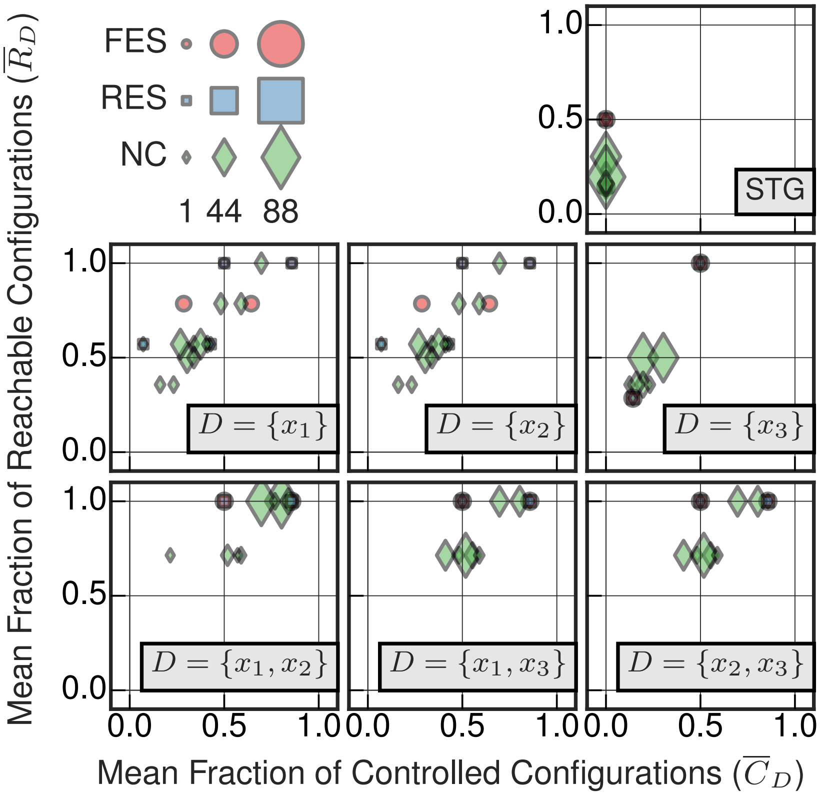

Figure S14: The average fraction of reachable configurations $\overline{R}$ and the average fraction of controllable configurations $\overline{C}$ for the full ensemble of 256 BN with structure given by the $N = 3$ variable CoRegulating network motif shown in Figure S1G, as controlled by the driver variable sets $D \equiv \{x_1\}, \{x_2\}, \{x_3\}, \{x_1, x_2\}, \{x_1, x_3\}, \{x_2, x_3\}$. There are 40 networks in the FES subset shown in red circles, 16 networks in the RES subset shown in blue squares, and 200 networks in the NC subset shown in green diamonds; the area of the object corresponds to the number of networks. Both SC and MDS methods predict $D \equiv \{x_1\}, \{x_2\}$ is sufficient to fully control the system dynamics.

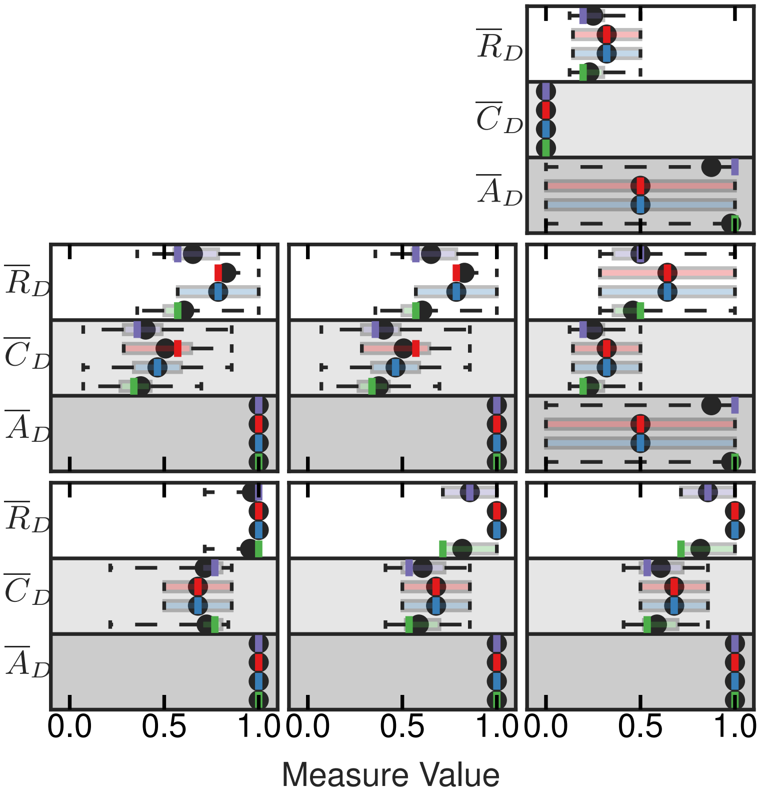

Figure S15: Boxplots summarizing the controllability measures for the CoRegulating network motif. These population statistics are shown for the Full Ensemble (purple), FES (red), and NC (green) subsets. The mean values for each measure and subset are denoted by black dots, the median values for each measure and subset are shown by the appropriately colored solid line, the box illustrates the interquartile range, and the whiskers denote the minimum and maximum values. Subplots correspond to the same driver variable sets as shown in Figure S14.

S2.3.8 BiParallel Motif

The $N = 4$ variable BiParallel network motif, shown in Figure S1H, is frequently found at a high statistical frequency in neural networks, food webs, and electrical circuits [23]. The BiParallel network motif also provides an example of an internal dilation, one of several classifications for driver variables uncovered by the SC methodology [5]. The full ensemble consists of 256 BN with 40 in the FES subset, 16 in the RES subset, and 200 in the NC subset. Both the SC and MDS methods predict two variables are sufficient to fully control the system dynamics, either $D \equiv \{x_1, x_2\}$ or $D \equiv \{x_1, x_3\}$. MDS also posits $D \equiv \{x_1, x_4\}$ as another possible driver variable set. The control profiles in Figure S16 show that the first two driver variable set are capable of fully controlling 112 networks; these include all of the members of the FES and RES subsets. However, the third driver variable set proposed by MDS is not capable of fully controlling any of the BN in the full ensemble. As shown by the population statistics in Figure S17, as long as variable x_1 is in the driver variable subset, the networks can be controlled between their 2 attractors.

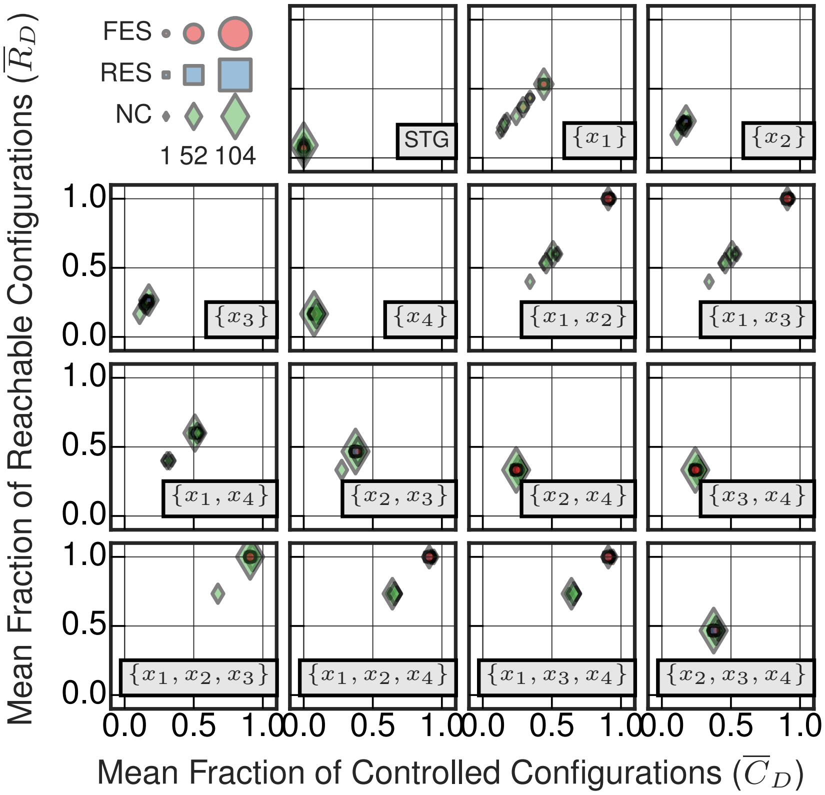

Figure S16: The average fraction of reachable configurations $\overline{R}$ and the average fraction of controllable configurations $\overline{C}$ for the full ensemble of 256 BN with structure given by the $N = 4$ variable BiParallel network motif shown in Figure S1H as controlled by the driver variable sets $D \equiv \{x_1\}, \{x_2\}, \{x_3\}, \{x_4\}, \{x_1, x_2\}, \{x_1, x_3\}, \{x_1, x_4\}, \{x_2, x_3\}, \{x_2, x_4\}, \{x_3, x_4\}, \{x_1, x_2, x_3\}$, $\{x_1, x_3, x_4\}, \{x_2, x_3, x_4\}$. There are 40 networks in the full effective structure (FES) subset shown in red circles, 16 networks in the reduced effective structure (RES) subset shown in blue squares, and 200 networks in the NC subset shown in green diamonds; the area of the object corresponds to the number of networks. Both SC and MDS methods predict $D \equiv \{x_1, x_2\}, \{x_1, x_3\}$ are equivalent and sufficient to fully control the system dynamics. MDS also predicts $D \equiv \{x_1, x_4\}$ can control network dynamics.

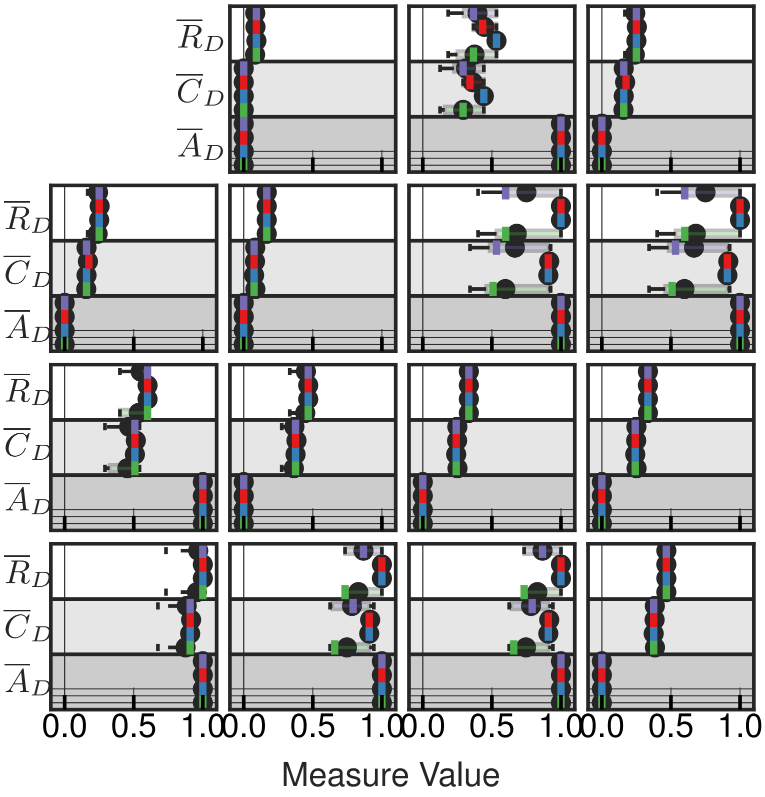

Figure S17: Boxplots summarizing the controllability measures for the CoRegulating network motif. These population statistics are shown for the Full Ensemble (purple), FES (red), and NC (green) subsets. The mean values for each measure and subset are denoted by black dots, the median values for each measure and subset are shown by the appropriately colored solid line, the box illustrates the interquartile range, and the whiskers denote the minimum and maximum values. Subplots correspond to the same driver variable sets as shown in Figure S16.

S2.3.9 BiFan Motif

The BiFan motif is the smallest example from the family of motifs known as dense overlapping regions or multi-input motifs [25], commonly found in gene regulatory networks and associated with a function similar to gate-arrays. It has already been the subject of an investigation into the correspondence between structure and dynamics for gene regulatory networks when ordinary differential equations are used to model biochemical interactions [29]. The structural network for the BiFan motif is shown in Figure S1I. There are 256 BN in the full ensemble; 100 networks are in the FES subset, 96 networks are in the RES subset, and 60 networks are in the NC subset. Both the SC and MDS methods predict $D \equiv \{x_1, x_2\}$ is sufficient to fully control the system dynamics. However, as the control profiles in Figure S18 show, this driver variable set is only capable of fully controlling 24 networks, all of which are members of the RES subset. The majority of networks with the BiFan structure require three variables to the be manipulated to render them fully controlled. Controlling these two variables is sufficient to control every network between its 4 attractors ($\overline{A} = 1.0$).

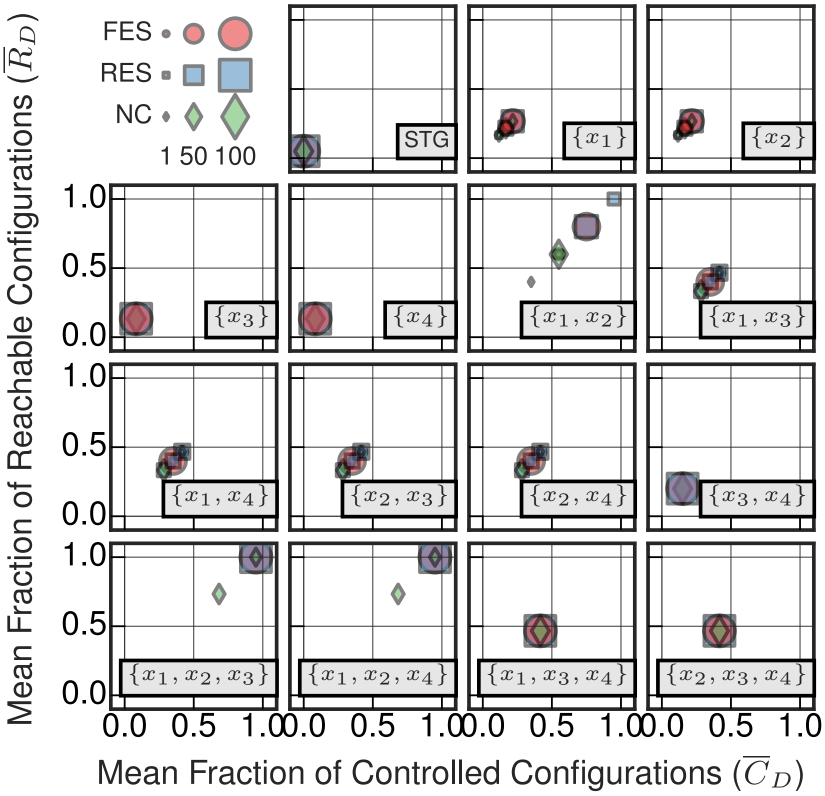

Figure S18: The average fraction of reachable configurations $\overline{R}$ and the average fraction of controllable configurations $\overline{C}$ for the full ensemble of 256 BN with structure given by the $N = 4$ variable BiFan network motif shown in Figure S1I, as controlled by the driver variable sets $D \equiv \{x_1\}, \{x_2\}, \{x_3\}, \{x_4\}, \{x_1, x_2\}, \{x_1, x_3\}, \{x_1, x_4\}, \{x_2, x_3\}, \{x_2, x_4\}, \{x_3, x_4\}, \{x_1, x_2, x_3\}, \{x_1, x_3, x_4\}, \{x_2, x_3, x_4\}$. There are 100 networks in the FES subset shown in red circles, 96 networks in the RES subset shown in blue squares, and 60 networks in the NC subset shown in green diamonds; the area of the object corresponds to the number of networks. Both SC and MDS methods predict $D \equiv \{x_1, x_2\}$ is sufficient to fully control the system dynamics.

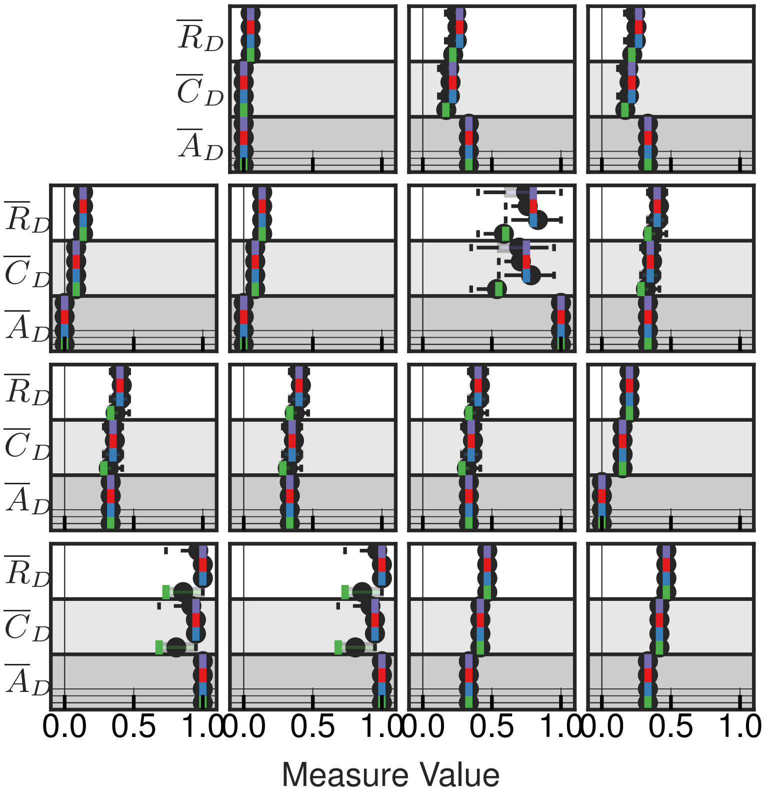

Figure S19: Boxplots summarizing the controllability measures for the BiFan network motif. These population statistics are shown for the Full Ensemble (purple), FES (red), and NC (green) subsets. The mean values for each measure and subset are denoted by black dots, the median values for each measure and subset are shown by the appropriately colored solid line, the box illustrates the interquartile range, and the whiskers denote the minimum and maximum values. Subplots correspond to the same driver variable sets as shown in Figure S18.

S2.3.10 Dominated Loop Motif

The $N = 4$ variable Dominated Loop network motif, shown in Figure S1J, is frequently overrepresented in neural networks, food webs, and electrical circuits [23]. The full ensemble consists of 4096 BN with 1000 in the FES subset, 1744 in the RES subset, and 1352 in the NC subset. Both the SC and MDS methods predict one variable is sufficient to full control the network dynamics, $D \equiv \{x_1\}$. However, as the control profiles in Figure S20 show, this driver variable is only capable of fully controlling 420 networks (96 FES and 324 RES). The population statistics in Figure S21, as long as variable x_1 is in the driver variable subset, the networks in the NC subset can be controlled between their 2 attractors. However, the RES subset has networks which contain $2, 3, 4, 5, 6$, and 8 attractors, while the FES subset has networks which contain $2, 3, 5, 6$ attractors. For both of these subsets, interventions to $D \equiv \{x_1\}$ results in full attractor control only when the networks which have 2 attractors. To render the entire ensemble of networks fully controlled between attracts, x_1 and another variable must be manipulated.

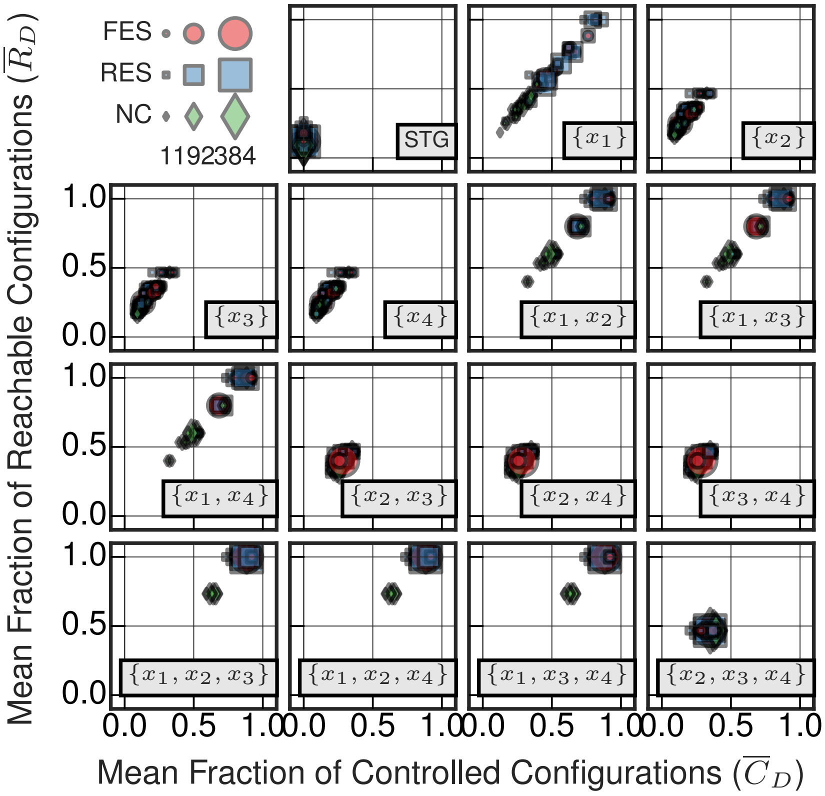

Figure S20: The average fraction of reachable configurations $\overline{R}$ and the average fraction of controllable configurations $\overline{C}$ for the full ensemble of 4096 BN with structure given by the $N = 4$ variable Dominated Loop network motif shown in Figure S1J, as controlled by the driver variable sets $D \equiv \{x_1\}, \{x_2\}, \{x_3\}, \{x_4\}, \{x_1, x_2\}, \{x_1, x_3\}, \{x_1, x_4\}, \{x_2, x_3\}, \{x_2, x_4\}, \{x_3, x_4\}, \{x_1, x_2, x_3\}$, $\{x_1, x_3, x_4\}, \{x_2, x_3, x_4\}$. There are 1000 networks in the FES subset shown in red circles, 1744 networks in the RES subset shown in blue squares, and 1352 networks in the NC subset shown in green diamonds; the area of the object corresponds to the number of networks. Both SC and MDS methods predict $D \equiv \{x_1\}$ is sufficient to fully control the system dynamics.

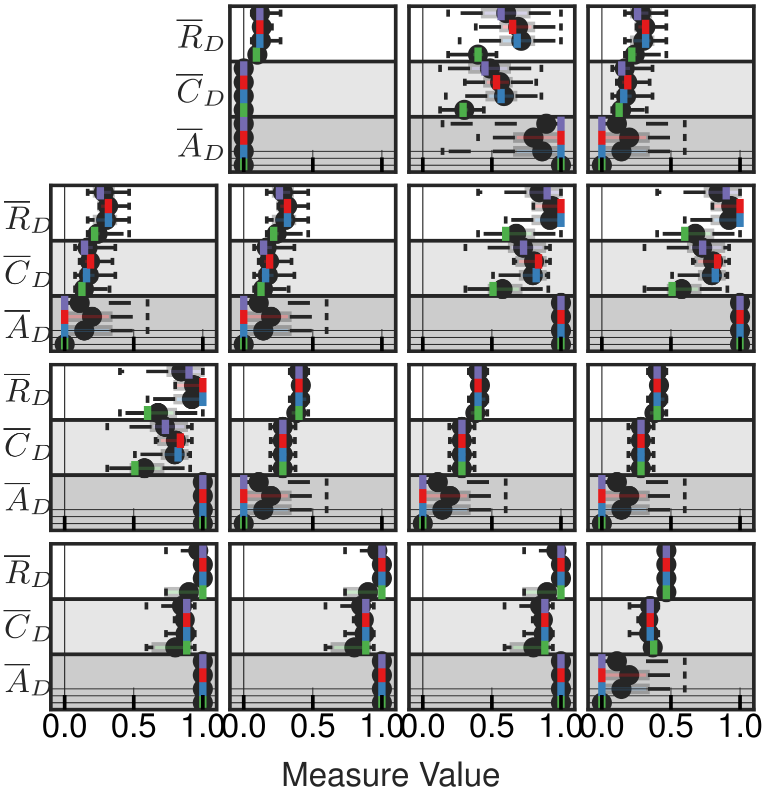

Figure S21: Boxplots summarizing the controllability measures for the Dominated Loop network motif. These population statistics are shown for the Full Ensemble (purple), FES (red), and NC (green) subsets. The mean values for each measure and subset are denoted by black dots, the median values for each measure and subset are shown by the appropriately colored solid line, the box illustrates the interquartile range, and the whiskers denote the minimum and maximum values. Subplots correspond to the same driver variable sets as shown in Figure S20.

S3 Biological Networks

S3.1 *Drosophila melanogaster* Segment Polarity Boolean Network

During the early ontogenesis of the fruit fly, body segmentation occurs [30]. The specification of adult cell types of the body segments is controlled by a sequential hierarchy of a few genes [31]. An ordinary differential equation model of this process, built from kinetic details of pairwise interactions, suggested that the regulatory network of segment polarity genes in *Drosophila melanogaster* is largely controlled by external inputs and is robust to changes to its internal kinetic parameters [32]. The Albert and Othmer BN version of this model [30] is capable of predicting the steady state patterns experimentally observed in wild-type and mutant embryonic development with significant accuracy. We analyze the single-cell segment polarity network (SPN) that regulates 17 gene and protein variables. This is a smaller version of the original 4-cell parasegment model, whose state-space can be fully enumerated [33] thus allowing precise understanding of how control ensues. This single cell model is represented by the structural network reproduced in Figure S22, where each variable's state follows the dynamical trajectories specified by synchronous updating of the logical transition functions shown in Table S1. There are 10 steady-state attractors of the system dynamics which constitute the wild-type and mutant expression patterns. The SPN is classified with full effective structure (FES).

It has been previously shown that segment polarity regulation, as modeled by the SPN, is highly controlled by the upstream value of the Sloppy Pair Protein (SLP) and the extra-cellular signals of the Hedgehog and Wingless proteins from neighboring cells nhh/nHH and nWG [30]. Indeed, when a single bit-flip perturbation of one or more of these three input nodes is allowed, it is known that dynamics can be controlled from any attractor to almost every other attractor, though not all [21]. The control portrait of this model (main text Figure 5) demonstrates that these three biochemical species are actually capable of fully controlling the dynamics from any attractor to any other attractor when we assume that any number of perturbations to the driver variables (e.g. repeated bit-flips of a variable) is possible. This means that under the same perturbation assumptions of structural control methods, the state of all intra-cellular chemical species is completely irrelevant for attractor control in this model. Cellular control of segment polarity regulation in the SPN is fully achieved by the upstream value of SLP and the extra-cellular nhh/nHH and nWG signals (driver set $\mathcal{S}0$ in main text Figure 5). The full control of dynamics from input variables (upstream and external proteins in the biochemical context) is to be expected in segment polarity regulation since it is a highly orchestrated developmental process where upstream signals control downstream dynamics.

Structural controllability (SC) analysis determines that the SPN is completely controllable from interventions applied to $|D| = 4$ four driver variables. A full enumeration of all possible maximum matchings reveals that there are four driver variable sets which

Node	Logic
SLP	-
wg	(CIA ∧ SLP ∧ ¬ CIR) ∨ (*wg* ∧ (CIA ∨ SLP) ∧ ¬ CIR)
WG	*wg*
en	nWG ∧ ¬ SLP
EN	*en*
hh	EN ∧ ¬ CIR
HH	*hh*
ptc	CIA ∧ ¬ EN ∧ ¬ CIR
PTC	*ptc* ∨ (PTC ∧ ¬ n*hh*/nHH)
PH	PTC ∧ n*hh*/nHH
SMO	¬ PTC ∨ n*hh*/nHH
ci	¬EN
CI	*ci*
CIA	CI ∧ (¬ PTC ∨ n*hh*/nHH)
CIR	CI ∧ PTC ∧ n*hh*/nHH
nWG	-
n*hh*/nHH	-

Table S1: The logical rules for the 17 node Boolean network of the single-cell model for segment polarity in *Drosophila melanogaster*.

satisfy the conditions of SC:

$$\begin{aligned}
\mathcal{S}1 &= \{\text{SLP, nWG, nhh/nHH, PH}\},\\
\mathcal{S}2 &= \{\text{SLP, nWG, nhh/nHH, CIA}\},\\
\mathcal{S}3 &= \{\text{SLP, nWG, nhh/nHH, SMO}\},\\
\mathcal{S}4 &= \{\text{SLP, nWG, nhh/nHH, CIR}\}.
\end{aligned} \tag{2}$$

According to the SC theory, these subsets are sufficient for fully controlling the BN from any configuration to another, yet, as demonstrated in the main text Figure 5, none of them is capable of fully controlling the network dynamics.

On the other hand, minimum Dominated set (MDS) analysis suggests that a minimum of $|D| = 7$ driver variables control the system dynamics. The full enumeration of the possible Dominated sets uncovers 8 equivalent sets shown in Table S2.

As discussed in the main text, none of these proposed sets are capable of fully controlling the system dynamics. Indeed, the maximum $\overline{R}$ value attained by the SC driver variables is $\overline{R} \approx 0.07$ while the MDS driver variables attain a maximum $\overline{R} \approx 0.31$. Interestingly, the maximum possible $\overline{R}$ achieved by a four driver variable subset was $\overline{R} \approx 0.12$ and not identified by the SC analysis. A full analysis of all 7-variable subsets was not conducted due to computational limitations.

Focusing our attention on the attractor control for the SPN reveals a different story. In

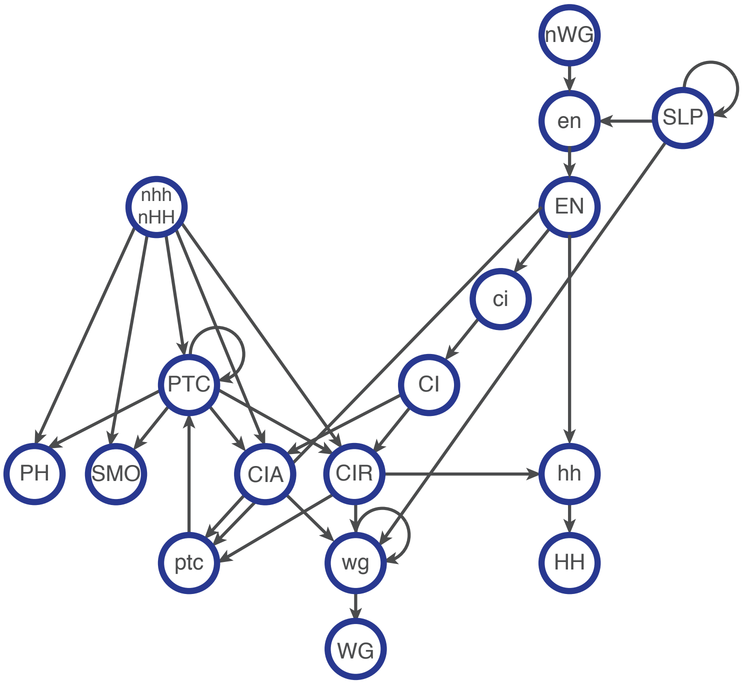

Figure S22: Structural graph of the SPN model. The 17 genes and proteins of the single-cell model for segment polarity in *Drosophila melanogaster*. Arrows indicate that the source variable is an input for the target variable's transition function.

this context, only three variables $\mathcal{S}0 = \{\text{SLP}, \text{nWG}, \text{nhh/nHH}\}$ are required to control the network from any of its attractors to any other attractor. These three variables are the root nodes of the structural network.

S3.2 *Saccharomyces cerevisiae* Cell Cycle Boolean Network

The eukaryotic cell cycle reflects the progression of the cell proliferation process, during which a single cell grows and divides into two daughter cells. The budding yeast *Saccharomyces cerevisiae* is a model eukaryotic organism which has been extensively investigated using both experimental and mathematical modeling techniques. While there are over 800 genes known to be involved in the budding yeast cell cycle process, many researchers have suggested that the process is sufficiently well approximated by only a handful of key regulators. Here, we use the 12 variable simplified Boolean model derived by Li et al. [34] and referred to as CCN. Allowing for all initial configurations of these 12 variables results in 11 steady-state attractors for the system dynamics (this differs from the 7 originally reported in [34] because the variable CellSize is also allowed to vary). Due to the transition function associated with the gene Swi5, this network is classified with reduced effective structure (RES).

Based solely on the structural network of CCN, structural controllability suggests that

Variables	$\overline{R}_D$	$\overline{C}_D$	$\overline{A}_D$
{SLP, nWG, nhh/nHH, EN, CI, WG, HH}	0.1210	0.1210	1.0
{SLP, nWG, nhh/nHH, EN, CI, wg, HH}	0.1434	0.1434	1.0
{SLP, nWG, nhh/nHH, EN, CI, WG, hh}	0.2156	0.2156	1.0
{SLP, nWG, nhh/nHH, EN, ci, WG, HH}	0.2156	0.2156	1.0
{SLP, nWG, nhh/nHH, EN, ci, wg, HH}	0.2351	0.2351	1.0
{SLP, nWG, nhh/nHH, EN, CI, wg, hh}	0.3132	0.3131	1.0
{SLP, nWG, nhh/nHH, EN, ci, wg, hh}	0.2351	0.2351	1.0
{SLP, nWG, nhh/nHH, EN, ci, WG, hh}	0.3132	0.3131	1.0

Table S2: The 8 equivalent sets determined by MDS analysis and their respective $\overline{R}_D$, $\overline{C}_D$ and $\overline{A}_D$ values.

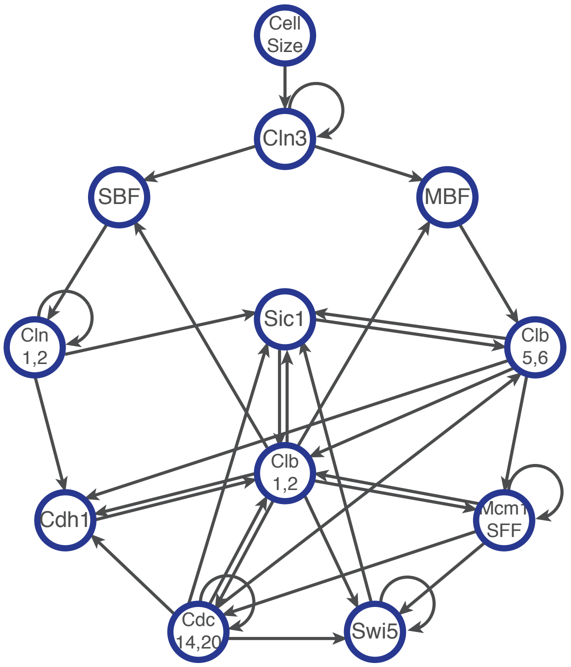

Figure S23: Structural graph of the 12 gene Boolean network model for the *Saccharomyces cerevisiae* cell cycle. Arrows indicate that the source variable is an input for the target variable's transition function.

the network is fully controllable from interventions applied to only one variable:

$$Y0 = \{CellSize\}. \tag{3}$$

However, our analysis found that this node is insufficient to fully control the network dynamics. MDS analysis identifies that $|D| = 4$ driver variables are required to control

the system, of which there are 8 possible sets:

$$\begin{aligned}
\mathcal{Y}1 &= \{\text{CellSize, SBF, Clb1/2, Cdc14,20}\},\\
\mathcal{Y}2 &= \{\text{CellSize, Cln1/2, Clb1/2, Cdc14,20}\},\\
\mathcal{Y}3 &= \{\text{CellSize, SBF, MBF, Clb1/2}\},\\
\mathcal{Y}4 &= \{\text{CellSize, SBF, Cln5/6, Clb1/2}\},\\
\mathcal{Y}5 &= \{\text{CellSize, SBF, Sic1, Clb1/2}\},\\
\mathcal{Y}6 &= \{\text{CellSize, SBF, Clb1/2,Cln1/2}\},\\
\mathcal{Y}7 &= \{\text{CellSize, Cln1/2, Cln5/6, Clb1/2}\},\\
\mathcal{Y}8 &= \{\text{CellSize, Cln1/2, Sic1, Clb1/2}\}.
\end{aligned} \tag{4}$$

As discussed in the main text, none of these driver variables sets were sufficient to render the network fully controllable. In fact, the driver sets predicted by MDS lead to values of both $\overline{A}_D$ and $\overline{R}_D$ that are essentially random, demonstrating once again that predictions with equivalent support from the point of view of the structure-only theories lead to widely different amounts of real controllability. In other words, structure-only analysis yields contradictory results vis a vis the controllability of the actual model dynamics.

Enumeration of all possible subsets of variables in the CCN revealed that the network can be controlled from each of its 11 attractors to each of the other attractors using interventions on a minimum of four variables, of which there are three equivalent driver variable sets (shown from left to right in Figure 6 from the main text):

$$\begin{aligned}
\mathcal{Y}9 &= \{\text{CellSize, SBF, MBF, Cdh1}\},\\
\mathcal{Y}10 &= \{\text{CellSize, SBF, MBF, Sic1}\},\\
\mathcal{Y}11 &= \{\text{CellSize, SBF, Cln5/6, Cdh1}\}.
\end{aligned} \tag{5}$$

Neither SC nor MDS predict those specific driver sets, which ultimately provide the most useful form of control in such systems. Arguably, in this case, the control prediction of SC is more off the mark than that of MDS since the attractor dynamics of the CCN is not even close to being controlled by a single variable.

The maximum $\overline{R}_D \approx 0.69$ is considerably larger than what was attained for the SPN model, but still quite far from the predicted full controllability (which would require $\overline{R}_D = 1.0$). When compared to the SPN, there are several striking features of this controllability profile. The first is the lack of striated bands associated with distinct input variables. Instead, the cloud of points reflects the huge variability in the effect of variable control. Another interesting feature is the lack of points around $\overline{A} = 0.0$. This indicates the propensity for manipulation of any variable to drive the network to a new attracting state. Inspection of the CAGs themselves reveals that interventions tend to transition the network into the one large basin of attraction associated with the biological stationary state, while it is relatively less common to see a transition out of this basin.

Even though the CCN is comprised of 12 variables and the SPN is comprised of 17 variables, only 3 variables are needed to fully control the attractor dynamics of the latter,

but at least 4 variables are required to attain the same control of the CCN. In contrast, a substantially smaller proportion of the total configurations are reachable by interventions to 4 variables in the SPN as compared to the CCN. This is coherent with the intent of each model; whereas control of attractors representing wild type and mutant cell phenotypes is the goal of the SPN, robustness of the cell-cycle attractor is the most salient feature of the CCN. To achieve attractor control, the SPN relies on a set of 3 (external) master controllers that are sufficient to switch dynamics to and from any attractor basin, but most configurations in these basins remain unreachable via the control of these 3 variables — controlling additional variables is necessary to reach a large proportion of the configurations, or the system needs to be initiated on them (garden-of-Eden configurations). The robustness of the wild type attractor of the CCN, on the other hand, relies on a very large basin of attraction which contains a large proportion of the total number of possible configurations [33]. Thus, control interventions tend to fall back on this basin sweeping a large proportion of configurations in the ensuing dynamics. This demonstrates that attractor control is not necessarily correlated with configuration control; the former can be fully achieved without much control ability of the latter form, as is the case of the SPN model. This is also observed in the CCN model, where we find driver sets of 4 variables with large attractor control, but relatively small configuration control and vice versa (see main text Figure 6A).

Most importantly for the purposes of our analysis is the fact that a structure-only analysis of controllability fails to differentiate these dynamical aspects of the models. Moreover, both structure-only analyses are completely wrong about how control actually operates. To summarize, SC (MDS) predicts that 4 (7) and 1 (4) variables are needed to fully control the SPN and the CCN, respectively. In reality, the predicted variables are very far from being able to achieve full control. If we consider solely attractor control, the same models in reality require 3 and 4 variables, respectively. Therefore, for the SPN model, both structure-only models overshoot the required variables (even though they include the 3 control variables since they are input variables in the interaction graph). For the CCN model, SC undershoots its prediction missing 3 additional required variables for attractor control, whereas MDS picks the correct number of variables but not the actual 4 variables capable of controlling attractor dynamics — indeed, the MDS predictions are essentially random from the attractor control perspective, given all the possible sets of 4 driver variables (see main text Figure 6A).

S3.3 *Arabidopsis thaliana* Floral Structure Boolean Network

During the development of the flower in angiosperms, cells are partitioned into four groups which eventually become the floral organs. The fate of these cells is determined by a network of interacting genes. Here, we use the 15 variable Boolean network underlying the cell-fate determination during floral organ specification in the flowering plant *Arabidopsis thaliana* [35, 36]. The network structure is shown in Figure S24 and is classified with reduced effective structure (RES) due to the transition function associated with the

gene LFY.

There are several interesting characteristics to the control profile of this system when controlled by all driver variable subsets of sizes $1 - 4$ shown in Figure S25. Firstly, the maximum $\overline{R}_D \approx 0.0544$ is smaller than that seen for both the SPN and CCN. Second, it is also interesting to note that no combination of four driver variables is capable of controlling the network between all of its attractors, the maximum $\overline{A}_D \approx 0.74$. Third, while the network structure has three root variables (as was the case for the SPN), labeled here as:

$$\mathcal{T}0 = \{LUG, CLF, UFO\} \tag{6}$$

interventions to these three variables endow the system with the same controllability as a random set of three variables for both $\overline{R}_D$ and $\overline{A}_D$.

The structural control methodologies similarity fail to identify the best subsets to control the network dynamics. Based solely on the structural network, SC suggests that the network is fully controllable from interventions applied to a 4 variable set, either:

$$\begin{aligned} \mathcal{T}1 &= \{LUG, CLF, UFO, EMF1\} \\ \mathcal{T}2 &= \{LUG, CLF, UFO, SEP\} \end{aligned} \tag{7}$$

However, our analysis found that neither of these two nodes are sufficient to fully control the network dynamics. MDS analysis identifies that at least 6 driver variables are required to control the system, of which there is only one possible set given in Table S3, which likewise cannot fully control the network dynamics.

The fact that the root (input) variables are insufficient to fully determine the attractor dynamics of the system mirrors the actual biology of the cell-fate determination process [36]. For example, the floral specification process is dependent on several earlier processes not modeled by the BN used here. During these processes, the genes *LFY* and *TFL1* are involved. At the same time, it is known that the gene *UFO* provides positional information that biases the floral specification; here this manifests itself as a relatively large $\mathcal{A}_D$ when *UFO* is controlled alone.

Variables	$\overline{R}_D$	$\overline{C}_D$	$\overline{A}_D$
{UFO, EMF1, LUG, CLF, TFL1, SEP}	0.1180	0.1179	0.4111

Table S3: The six driver variable set identified by MDS analysis and its respective $\overline{R}_D$, $\overline{C}_D$ and $\overline{A}_D$ values.

S4 Canalization and Boolean Network Control

We previously observed that in the set of contingent Boolean functions, there are *canalizing* functions [37, 38]. Their logic contains some redundancy in that the transition is

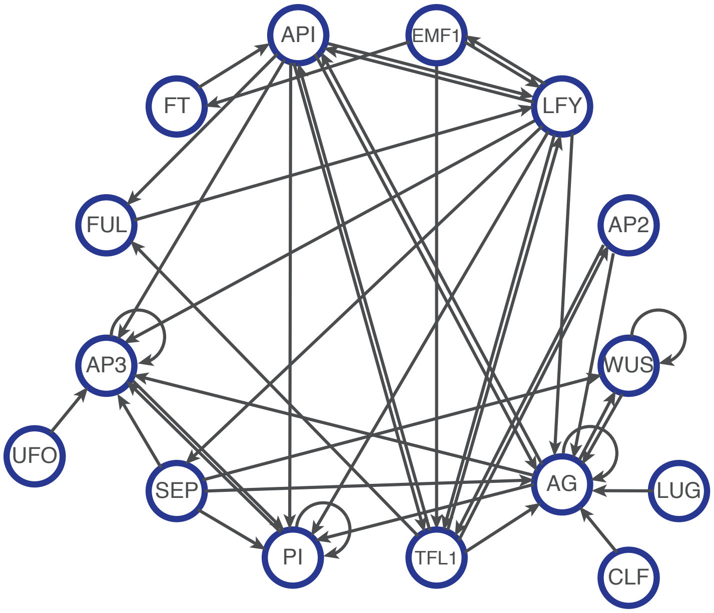

Figure S24: Structural graph of the 15 gene Boolean Network model for the cell-fate determination during floral organ specification in *Arabidopsis thaliana*. Arrows indicate that the source variable is an input for the target variable's transition function.

dictated only by the state of a subset of their inputs. When fully canalizing functions are present in a BN, not all of the edges in the structural graph contribute to the collective dynamics; there exists a subgraph that fully captures the dynamically relevant interactions, which we refer to as an *effective* structural graph [21, 39]. Moreover, most Boolean functions are *partially* canalizing [21, 38] whereby in some input conditions a subset of inputs is redundant, but in other conditions it is not. For instance, for the logical function AND, when one input is in state 0, the state of the other input is redundant. Indeed, for every class of Boolean functions of in-degree $k_i > 0$, there are only two non-canalizing functions (with full *effective connectivity* [21]); for example, for $k_i = 2$ the logical function XOR and its negation are the only two functions with full effective connectivity. This means that most edges in the underlying structural graph of a random BN are either entirely or partially redundant.

To gain greater insight about the role of canalization in determining the extent to which network structure can predict the controllability of multivariate Boolean dynamics. More specifically, we compared ensembles of networks with and without canalization in two different scenarios: the structural graph constrained by the *Saccharomyces cerevisiae* Cell Cycle Boolean Network (CCN) and structural graphs constrained by certain network motifs.

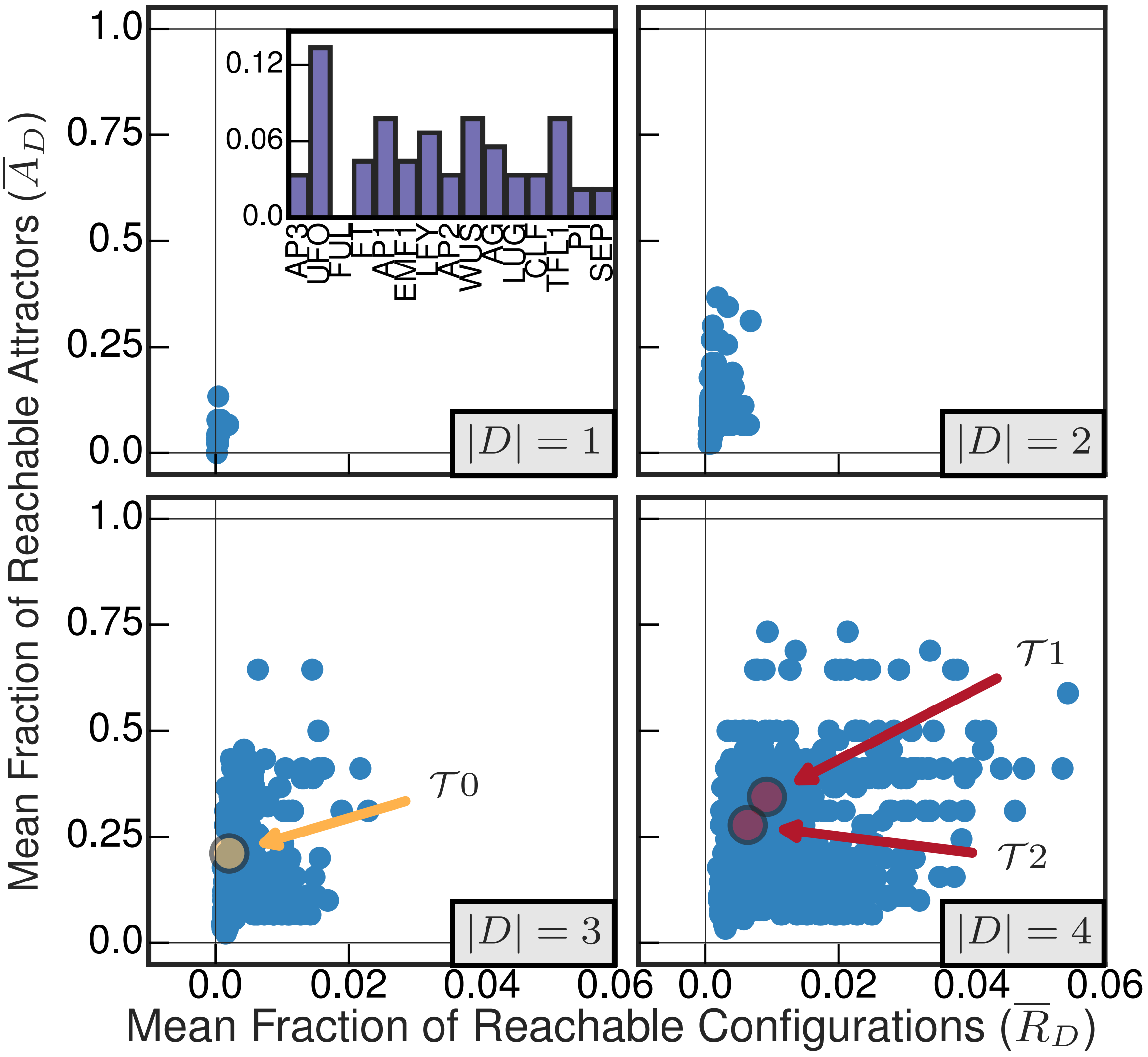

Figure S25: Control of the Boolean Network modeling the cell-fate determination during floral organ specification in the flowering plant *Arabidopsis thaliana* for all driver variable subsets of size $|D| = 1$, $|D| = 2$, $|D| = 3$, and $|D| = 4$. (inset) The mean fraction of reachable attractors $\overline{A}_D$ for each singleton driver variable set. The driver variable subsets predicted by structural controllability (SC) to fully control the network are highlighted in red and labeled $\mathcal{T}1$ and $\mathcal{T}2$. The three variable subset with all three root variables is highlighted in yellow and labeled $\mathcal{T}0$.

S4.1 Canalization in *Saccharomyces cerevisiae*

A sample of 50 networks was drawn from FEC and the system controllability was calculated for all driver variable sets of size $|D| = 1, 2, 3, 4$. The results of this calculation are presented in Table S4 as an average across the sample, as well as the BNs which were the most controlled and the least controlled on average. The corresponding calculations for the original CCN model are also shown for a comparison.

Even though all networks have the same structural graph, and all Boolean functions are without canalization, there is variability in the minimum number of driver variables which render the networks fully controllable. In the best case from the FEC ensemble, only 2 variables are need to fully control network dynamics ($\overline{R}_D = 1.0$) for which there are 3 equivalent sets (see Figure S26). However, in the worst case from the FEC ensemble, 3 variables are needed to fully control network dynamics ($\overline{R}_D = 1.0$) and there are 4 equivalent sets (see Figure S27).

Focusing on the specific predictions of the two structure-only methodologies considered here (SC and MDS), we see that even when there is the strongest correspondence between the structural graph and the dynamical interactions, both methods failed to accurately predict the controllability of the networks. This is illustrated by the control profiles for all driver variable subsets of sizes $1 - 4$ of the network with the best average control (Best FEC, Figure S26) and the network with the worst average control (Worst FEC, Figure S27) $\overline{R}_D$ averaged over all driver variable sets. Indeed, no networks from the FEC ensemble were fully controllable via interventions to only one driver variable as predicted by the SC analysis. While the specific driver variable predicted by SC ($D = \{\text{CellSize}\}$) consistently has the highest $\overline{R}_D$ of all singleton driver variable sets, there are always at least two other singleton driver variable sets which have higher $\overline{A}_D$ in every network from our sample ($D = \{\text{MBF}\}$ and $D = \{\text{Cdc14/20}\}$). On the other hand, the predictions of the MDS analysis overestimated the actual number of driver variable sets needed to fully control the networks in the FEC ensemble. Surprisingly, many networks in the FEC ensemble were fully controllable $\overline{R}_D = 1.0$ with interventions to only 2 driver variables, while all networks could be fully controlled by interventions to 3 driver variables. Looking at the specific driver variable sets uncovered by the MDS analysis, we see that it incorrectly identified some driver variable sets as able to fully control the dynamics, and missed many other driver variable sets of the same size which were actually able to fully control the network dynamics (seen by the large number of overlapping points in Figures S26, S27). Thus, even though SC undershoots and MDS overshoots the number of nodes necessary to attain full controllability, for the networks in the FEC ensemble, these predictions are closer to the real number of driver variables needed.

The sample of networks from FEC is fully controllable with interventions to fewer variables — and thus matching structure-only predictions of size of driver sets better; possibly because the effective structure is maximal (the same as the original underlying structure). In such a case, the structural interactions by which control can be exerted and propagated are maximized in comparison to the possible interactions that exist in the real CCN — which contains canalizing functions and thus possesses a smaller effective

structural graph. Indeed, the increase in controllability for BNs without canalization is related to both the increase of chaos in BNs with greater connectivity and the increase of robustness to perturbations exhibited by BNs with canalization [37, 40]. When there is no canalization, the effective connectivity of the network is larger and so we can expect more chaotic regimes [39]; in this case, smaller perturbations spread more easily such that all configurations are more reachable leading to an increase in the measures of controllability. When canalization arises (as in the real CCN model), we tend to observe the buffering of the effect of perturbations [41]; such robustness to perturbations implies a reduction in the ability to fully control the network [37].

It should be emphasized, however, that the actual control variables predicted by the structure-only theories are still mostly incorrect even for the FEC ensemble. This suggests that there are other factors which determine the actual controllability of BN dynamics but which are not captured by the structural interaction graphs. Such factors may depend on nonlinearity in the collective integration of multi-variate dynamics, as well as the exact patterns of canalized interactions. While the random sampling of the FEC ensemble shows that the absence of canalization leads to higher controllability, it is also known that canalizing functions can be engineered (by human-design or natural selection) such that control pathways are more effective (and simultaneously robust) in exerting attractor control in biological networks — e.g. the SPN model of drosophila embryological development contains canalized pathways modules highly effective at controlling dynamics [21].

S4.2 Canalization in Network Motifs

We look at a subset of the full ensembles constrained by network motifs examined in the main text and Section S2. As previously discussed in the main text and Section S2.2, the full ensemble of Boolean Networks was partitioned into three subsets: the non-contingent subset (NC), the reduced effective structure subset (RES), and the full effective structure subset (FES) based on the criteria that at least one of the transition functions was NC, RES or FES. Here, we focus on the smaller subsets of BNs such that all of the transition functions are NC or RES; denoted Pure NC (PNC) and Pure RES (PRES). We also eliminate all partially canalized functions from the FES subset. The resulting subset of BNs, denoted Pure FES (PFES), contains only those networks where all transition functions have full effective connectivity (for degree $k_i = 2$ this is only the XOR logic and its negation). As in the previous section (S4.1), the PFES subset contains networks with dynamics that are most accurately captured by their structural graphs in that every interaction plays an equal and maximal role in determining the variables' transitions. Since there are no PRES transition functions of degree $k_i = 1$, we limit this exploration to the 4 network motifs with variable degrees of $k_i = 0$ or $k_i = 2$: the Loop Motif with self-interactions (Figures S28 & S29), the CoRegulated Motif (Figures S30 & S31), the BiFan Motif (Figures S32 & S33), and the Dominated Loop Motif (Figures S34 & S35).

First, we focus on the results obtained for the PFES subset. Once again, both structure

Ensemble	Driver Variable Size	Mean $\overline{R}_D$	Mean $\overline{A}_D$	# $\overline{R}_D = 1.0$
FEC	1	0.119 (0.0275)	0.1851 (0.0441)	0 (0)
	2	0.2266 (0.0487)	0.2979 (0.06435)	1.5667 (1.1358)
	3	0.3282 (0.0620)	0.3969 (0.0744)	17.86 (9.7837)
	4	0.4351 (0.0959)	0.4920 (0.0943)	80.74 (33.7448)
CCN	1	0.0088	0.0985	0
	2	0.0317	0.1920	0
	3	0.0875	0.2824	0
	4	0.1817	0.3762	0
Best FEC	1	0.1434	0.2333	0
	2	0.2798	0.3758	3
	3	0.4021	0.4915	31
	4	0.5090	0.5836	127
Worst FEC	1	0.0717	0.1129	0
	2	0.1466	0.1975	0
	3	0.2305	0.2848	4
	4	0.3228	0.3746	32

Table S4: A comparison of the 50 networks sampled from the FEC with the original CCN, most controlled sample (Best FEC), and least controlled sample (Worst FEC). The mean for $\overline{R}_D$ and $\overline{A}_D$ are both found by averaging over all driver variable sets of a given size. The number of driver variables which rendered the networks fully controllable # $\overline{R}_D = 1.0$ is also shown. All of the FEC values are averaged over 50 samples with standard deviation given in parentheses.

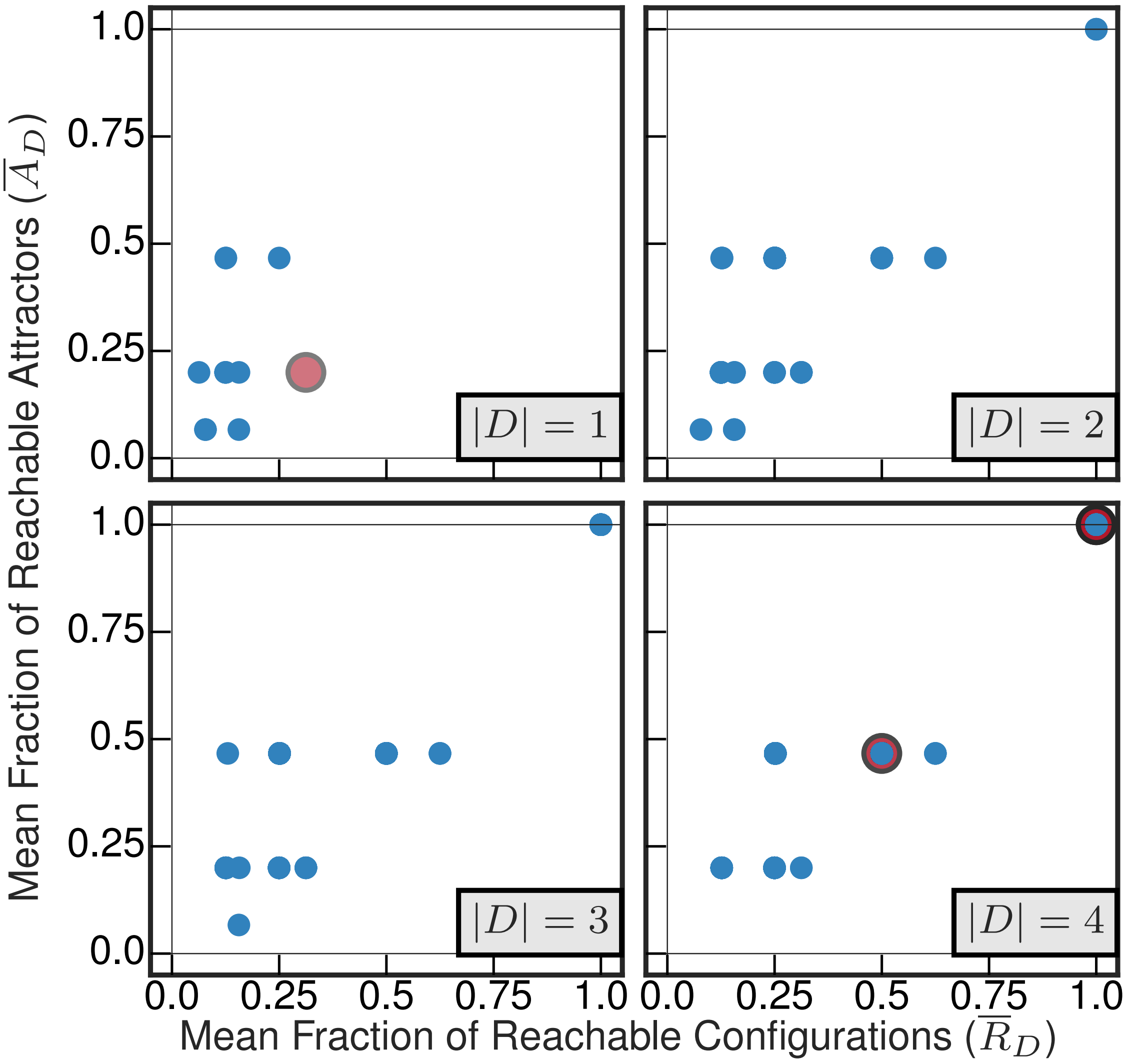

Figure S26: Control of an exemplar BN constrained by the CCN when all of the logic transitions functions are randomly assigned from the set with full effective connectivity for all driver variable subsets of size $|D| = 1$, $|D| = 2$, $|D| = 3$, and $|D| = 4$. This exemplar was identified as the network from the sample with the highest mean $\overline{R}_D$ for each driver variable size. The driver variable sets predicted by structure only methods to fully control the network are highlighted in red. There are 3 sets of $|D| = 2$, 31 of $|D| = 3$, and 127 of $|D| = 4$ which render the network fully controllable ($\overline{R}_D = 1.0$).

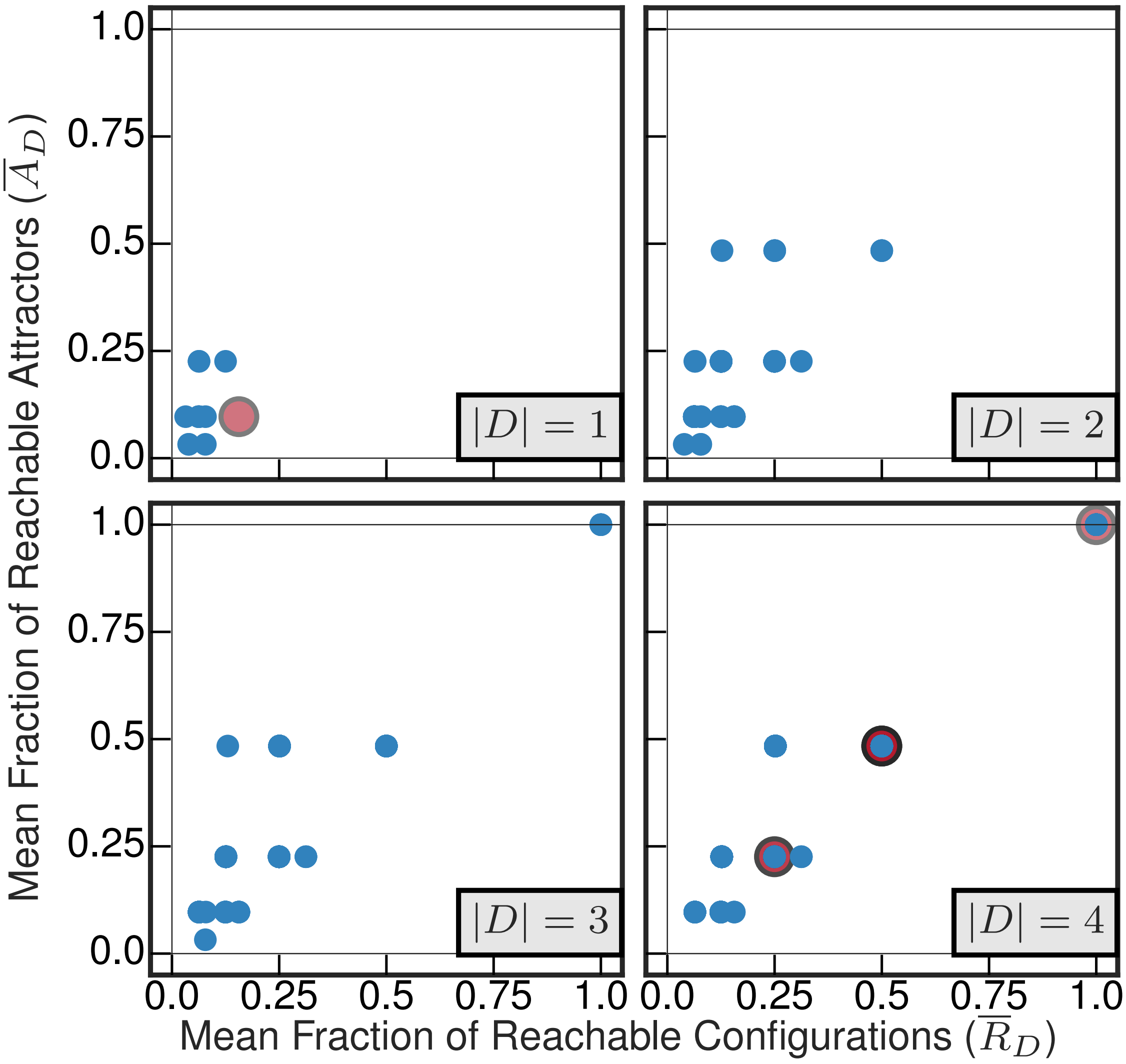

Figure S27: Control of an exemplar BN constrained by the CCN when all of the logic transitions functions are randomly assigned from the set with full effective connectivity for all driver variable subsets of size $|D| = 1$, $|D| = 2$, $|D| = 3$, and $|D| = 4$. This exemplar was identified as the network from the sample with the lowest mean $\overline{R}_D$ for each driver variable size. The driver variable sets predicted by structure only methods to fully control the network are highlighted in red. There are 0 sets of $|D| = 2$, 4 of $|D| = 3$, and 32 of $|D| = 4$ which render the network fully controllable ($\overline{R}_D = 1.0$).

only control methods failed to characterize the controllability of every BN instance. SC correctly identified the driver variable subset for only one of these Motif ensembles (the Loop with self-interactions), while many of the networks in the other three motifs' ensembles were not fully controlled by the SC predictions. The MDS analysis similarly failed to correctly identify the driver variable subsets which fully control the PFES networks for three of the four motifs, and overshoots the minimum number needed for the only case where it correctly identifies the subset: the Loop Motif with self-interactions. Therefore, even in these small network motifs for which the network dynamics are most accurately captured by their structural graphs, the structure only control methods fail to accurately predict the controllability of the system dynamics.

Second, we focus on a more detailed analysis for the PRES subset constrained by these 4 network motifs. BNs in the PRES subset have many interactions present in their structural graphs which are completely ignored by the actually dynamic transition functions. Interestingly, for every one of these motifs, there were some networks from the PRES subset which were accurately characterized by the structure only methods. In all of the cases where BNs from the PRES were fully controllable by a structure only method, their canalization resulted in reduced effective structures equivalent to chain motifs of 2, 3, or 4 variables as depicted in Figure S36. Thus, it appears canalization can enhance the accuracy of structure only control methodologies by reducing the effective dynamics to a linear chain of interacting variables.

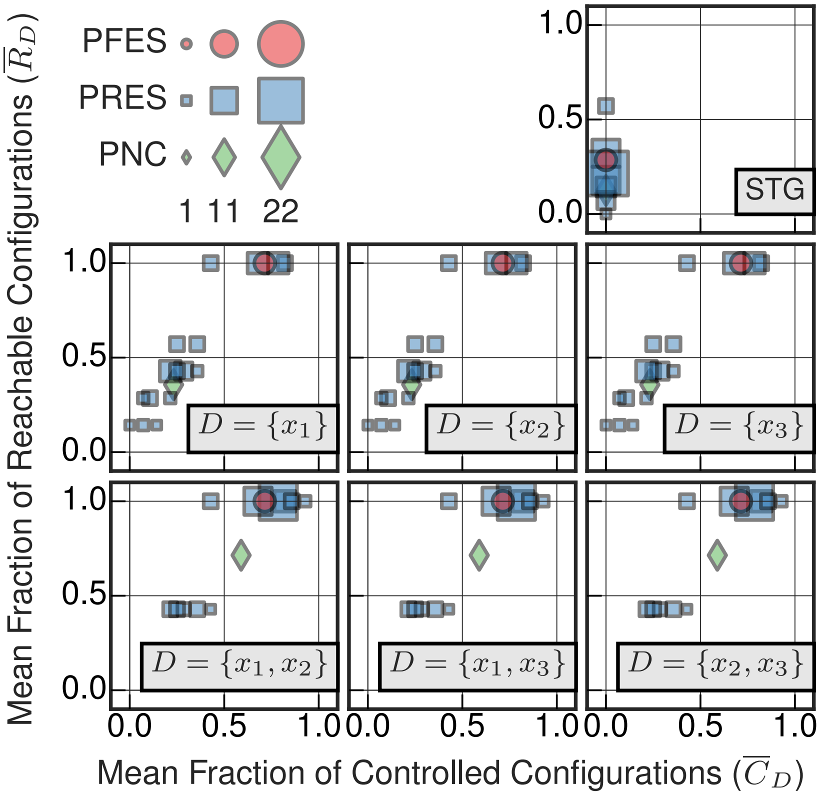

Figure S28: The average fraction of reachable configurations $\overline{R}$ and the average fraction of controllable configurations $\overline{C}$ for the ensemble of 140 pure BN with structure given by the $N = 3$ variable Loop network motif with self interactions, shown in Figure S1D, as controlled by the driver variable sets $D \equiv \{x_1\}, \{x_2\}, \{x_3\}, \{x_1, x_2\}, \{x_1, x_3\}, \{x_2, x_3\}$. There are 8 networks in the pure FES (PFES) subset shown in red circles, 64 networks in the pure RES (PRES) subset shown in blue squares, and 8 networks in the pure NC (PNC) subset shown in green diamonds; the area of the object corresponds to the number of networks. SC predicts any singleton set ($D \equiv \{x_1\}, \{x_2\}, \{x_3\}$) is sufficient to fully control the system dynamics, while MDS predicts any two variables ($D \equiv \{x_1, x_2\}, \{x_1, x_3\}, \{x_2, x_3\}$) are necessary.

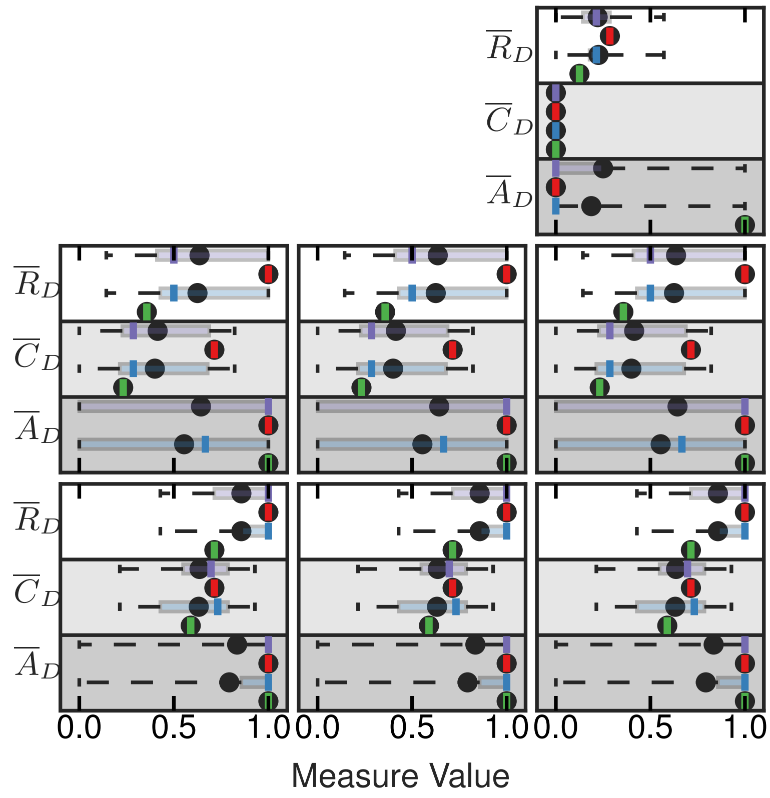

Figure S29: Boxplots summarizing the controllability measures for the Loop network motif with self interactions. These population statistics are shown for the full Pure Ensemble (purple), PFES (red), PRES (blue) and PNC (green) subsets. The mean values for each measure and subset are denoted by black dots, the median values for each measure and subset are shown by the appropriately colored solid line, the box illustrates the interquartile range, and the whiskers denote the minimum and maximum values. Subplots correspond to the same driver variable sets as shown in Figure S28.

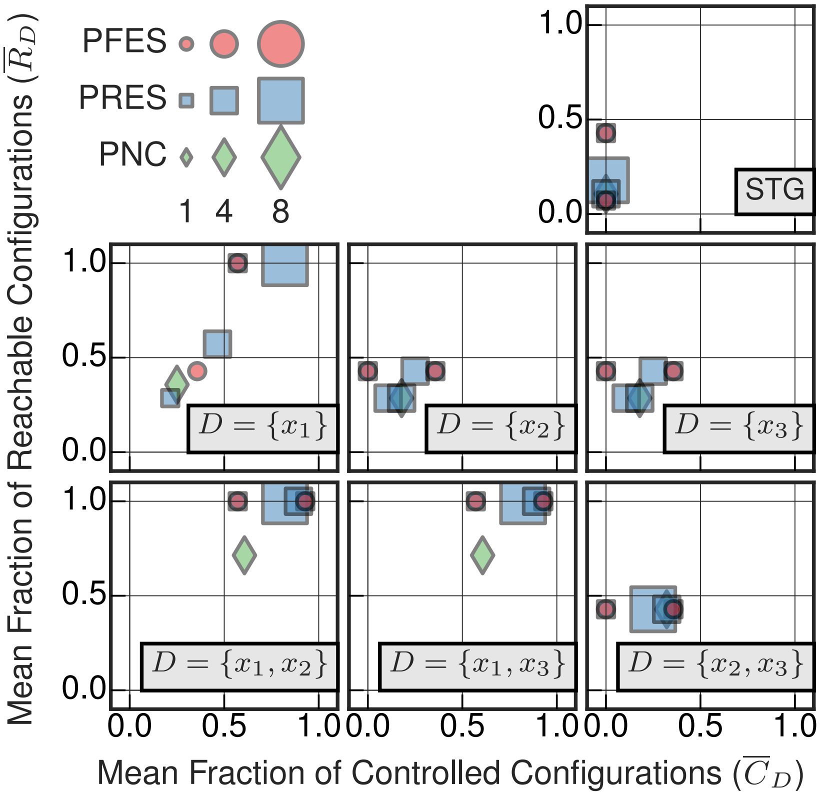

Figure S30: The average fraction of reachable configurations $\overline{R}$ and the average fraction of controllable configurations $\overline{C}$ for the ensemble of 140 pure BN with structure given by the $N = 3$ variable CoRegulated network motif shown in Figure S1F, as controlled by the driver variable sets $D \equiv \{x_1\}, \{x_2\}, \{x_3\}, \{x_1, x_2\}, \{x_1, x_3\}, \{x_2, x_3\}$. There are 4 networks in the pure FES (PFES) subset shown in red circles, 16 networks in the pure RES (PRES) subset shown in blue squares, and 4 networks in the pure NC (PNC) subset shown in green diamonds; the area of the object corresponds to the number of networks. Both SC and MDS methods predict $D \equiv \{x_1\}$ is sufficient to fully control the system dynamics.

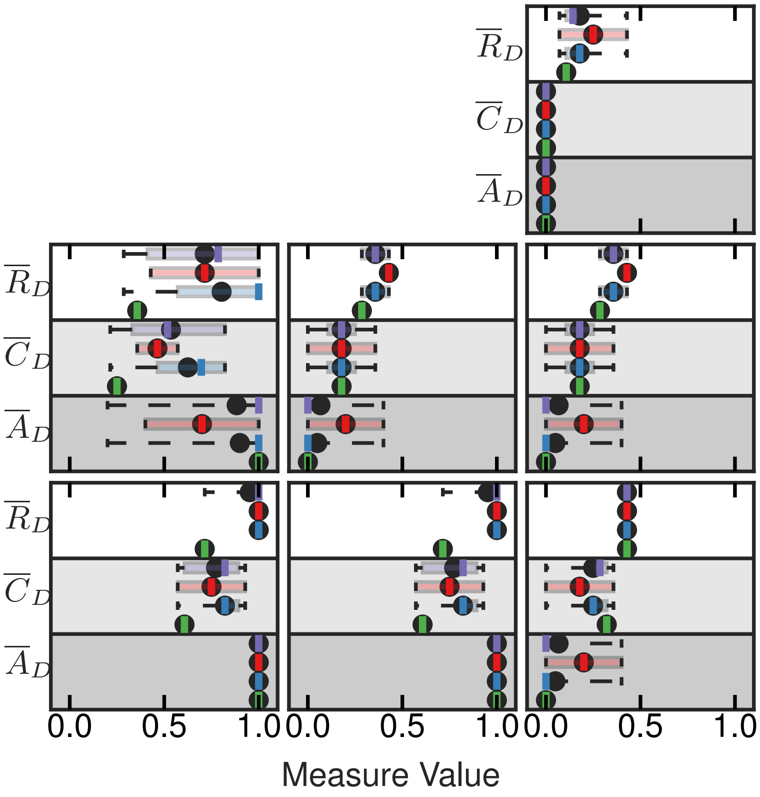

Figure S31: Boxplots summarizing the controllability measures for the CoRegulated network motif. These population statistics are shown for the full Pure Ensemble (purple), PFES (red), and PNC (green) subsets. The mean values for each measure and subset are denoted by black dots, the median values for each measure and subset are shown by the appropriately colored solid line, the box illustrates the interquartile range, and the whiskers denote the minimum and maximum values. Subplots correspond to the same driver variable sets as shown in Figure S30.

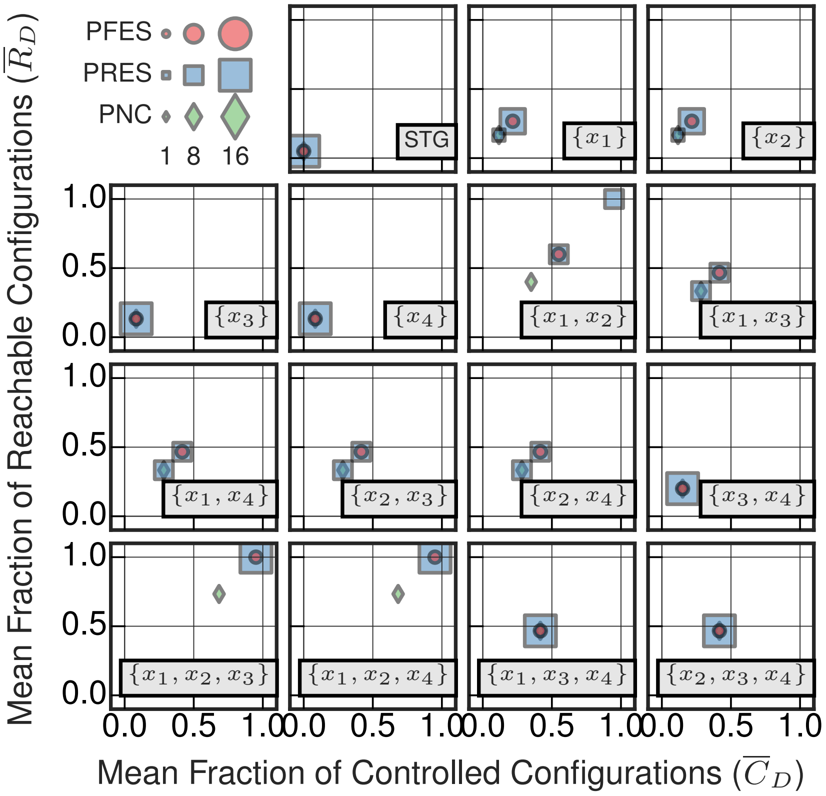

Figure S32: The average fraction of reachable configurations $\overline{R}$ and the average fraction of controllable configurations $\overline{C}$ for the ensemble of 120 pure BN with structure given by the $N = 4$ variable BiFan network motif shown in Figure S1I, as controlled by the driver variable sets $D \equiv \{x_1\}, \{x_2\}, \{x_3\}, \{x_4\}, \{x_1, x_2\}, \{x_1, x_3\}, \{x_1, x_4\}, \{x_2, x_3\}, \{x_2, x_4\}, \{x_3, x_4\}, \{x_1, x_2, x_3\}$, $\{x_1, x_3, x_4\}, \{x_2, x_3, x_4\}$. There are 4 networks in the pure FES (PFES) subset shown in red circles, 16 networks in the pure RES (PRES) subset shown in blue squares, and 4 networks in the pure NC (PNC) subset shown in green diamonds; the area of the object corresponds to the number of networks. Both SC and MDS methods predict $D \equiv \{x_1, x_2\}$ is sufficient to fully control the system dynamics.

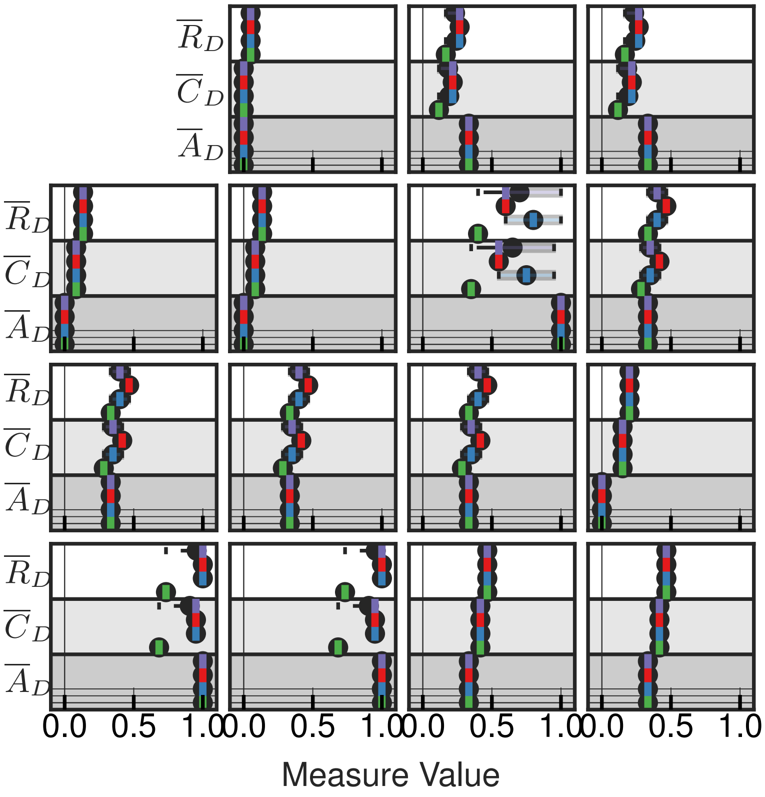

Figure S33: Boxplots summarizing the controllability measures for the BiFan network motif. These population statistics are shown for the full Pure Ensemble (purple), PFES (red), and PNC (green) subsets. The mean values for each measure and subset are denoted by black dots, the median values for each measure and subset are shown by the appropriately colored solid line, the box illustrates the interquartile range, and the whiskers denote the minimum and maximum values. Subplots correspond to the same driver variable sets as shown in Figure S32.

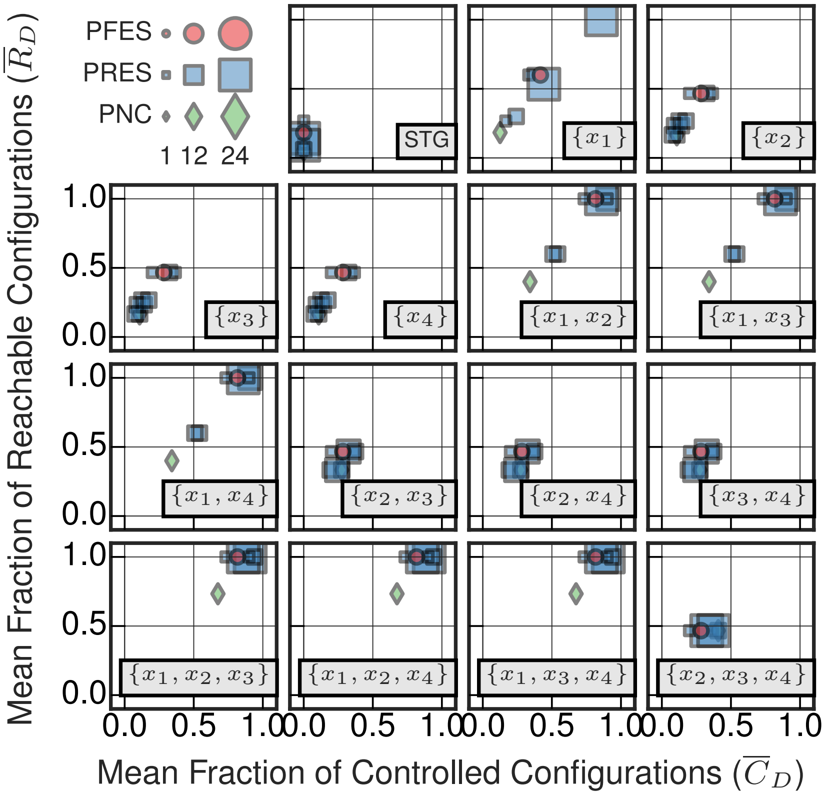

Figure S34: The average fraction of reachable configurations $\overline{R}$ and the average fraction of controllable configurations $\overline{C}$ for the ensemble of 1072 pure BN with structure given by the $N = 4$ variable Dominated Loop network motif shown in Figure S1J, as controlled by the driver variable sets $D \equiv \{x_1\}, \{x_2\}, \{x_3\}, \{x_4\}, \{x_1, x_2\}, \{x_1, x_3\}, \{x_1, x_4\}, \{x_2, x_3\}, \{x_2, x_4\}, \{x_3, x_4\}, \{x_1, x_2, x_3\}$, $\{x_1, x_3, x_4\}, \{x_2, x_3, x_4\}$. There are 8 networks in the pure FES (PFES) subset shown in red circles, 64 networks in the pure RES (PRES) subset shown in blue squares, and 8 networks in the pure NC (PNC) subset shown in green diamonds; the area of the object corresponds to the number of networks. Both SC and MDS methods predict $D \equiv \{x_1\}$ is sufficient to fully control the system dynamics.

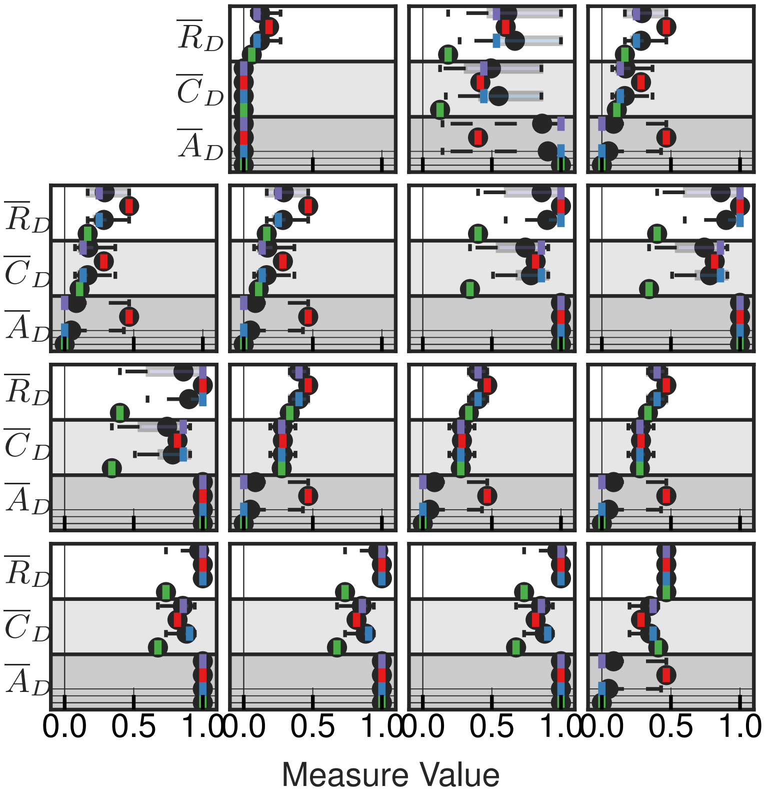

Figure S35: Boxplots summarizing the controllability measures for the Dominated Loop network motif. These population statistics are shown for the full Pure Ensemble (purple), PFES (red), PRES (blue), and PNC (green) subsets. The mean values for each measure and subset are denoted by black dots, the median values for each measure and subset are shown by the appropriately colored solid line, the box illustrates the interquartile range, and the whiskers denote the minimum and maximum values. Subplots correspond to the same driver variable sets as shown in Figure S34.

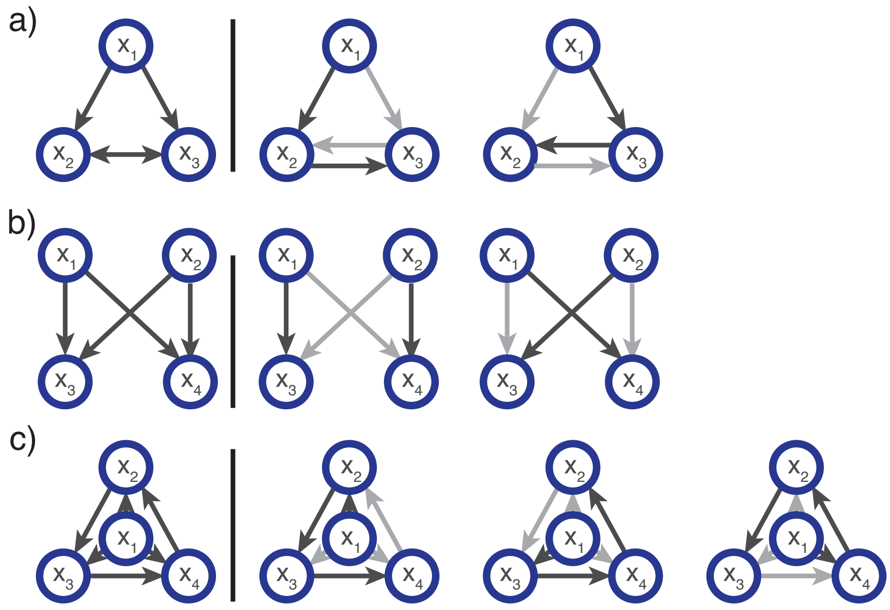

Figure S36: The a) Co-regulated, b) BiFan, and c) Dominated Loop network motifs and the reduced effective structure for networks in the PRES which were fully controllable as predicted by SC and MDS analysis. Light grey edges denote structural interactions which are completely ignored by the logical transition functions.

References

[1] Sontag, E. D. Mathematical control theory: deterministic finite dimensional systems. Springer, New York (1998).

[2] Dion, J.-M. . M., Commault, C. & Van Der Woude, J. Generic properties and control of linear structured systems: a survey. *Automatica* **39**, 1125–1144 (2003).

[3] Lin, C. Structural controllability. *IEEE Trans. Automat. Contr.* **19**, 201–208 (1974).

[4] Shields, R. W. & Pearson, J. B. Structural controllability of multiinput linear systems. *IEEE Trans. Automat. Contr.* **21**, 203–212 (1976).

[5] Ruths, J. & Ruths, D. Control profiles of complex networks. *Science* **343**, 1373–1376 (2014).

[6] Commault, C., Dion, J.-M. . M. & van der Woude, J. W. Characterization of generic properties of linear structured systems for efficient computations. *Kybernetika* **38**, 503–520 (2002).

[7] Liu, Y.-Y., Slotine, J.-J. & Barabási, A.-L. Controllability of complex networks. *Nature* **473**, 167–173 (2011).

[8] Hopcroft, J. E. & Karp, R. M. An $nn^5/2$ algorithm for maximum matchings in bipartite graphs. *SIAM Journal on computing* **2**, 225–231 (1973).

[9] Delpini, D. *et al.* Evolution of controllability in interbank networks. *Scientific Reports* **3** (2013).

[10] Meng, X., Xiang, W. & Wang, L. Controllability of train service network. *Mathematical Problems in Engineering* **2015** (2015).

[11] sterlund, T., Bordel, S. & Nielsen, J. Controllability analysis of transcriptional regulatory networks reveals circular control patterns among transcription factors. *Integrative Biology* **7**, 560–568 (2015).

[12] Egerstedt, M. Complex networks: Degrees of control. *Nature* **473**, 158–159 (2011).

[13] Nacher, J. C. & Akutsu, T. Dominating scale-free networks with variable scaling exponent: heterogeneous networks are not difficult to control. *New J. Phys.* **14**, 073005 (2012).

[14] Nacher, J. C. & Akutsu, T. Structural controllability of unidirectional bipartite networks. *Scientific Reports* **3** (2013).

[15] Vazquez, A. Optimal drug combinations and minimal hitting sets. *BMC systems biology* **3**, 81 (2009).

[16] Wuchty, S. Controllability in protein interaction networks. *Proc. Natl. Acad. Sci. USA U.S.A.* **111**, 7156–7160 (2014).

[17] Milenkovi, T., Memi\ vsevi, V., Bonato, A. & Pr\ vzulj, N. v. Dominating biological networks. *PloS One* **6**, e23016–e23016 (2011).

[18] Wang, B. *et al.* Diversified control paths: A significant way disease genes perturb the human regulatory network. *PLoS One* **10** (2015).

[19] Nacher, J. C. & Akutsu, T. Analysis of critical and redundant nodes in controlling directed and undirected complex networks using dominating sets. *Journal of Complex Networks* cnu029 (2014).

[20] Kant, M. M., Limouzy, V., Mary, A. & Nourine, L. *Enumeration of Minimal Dominating Sets and Variants*, 298–309 (Springer Berlin Heidelberg, 2011).

[21] Marques-Pita, M. & Rocha, L. M. Canalization and control in automata networks: body segmentation in Drosophila melanogaster. *PloS One* **8**, e55946 (2013).

[22] Quine, W. V. A Way to Simplify Truth Functions. *American Mathematical Monthly* **62**, 627–631 (1955).

[23] Milo, R. *et al.* Network Motifs: Simple Building Blocks of Complex Networks. *Sci. Signal.* **298**, 824–764 (2002).

[24] Yeger-Lotem, E. *et al.* Network motifs in integrated cellular networks of transcription-regulation and protein-protein interaction. *Proc. Natl. Acad. Sci. USA* **101**, 5934–5939 (2004).

[25] Alon, U. Network motifs: theory and experimental approaches. *Nat. Rev. Genet.* **8**, 450–461 (2007).

[26] Mangan, S. & Alon, U. Structure and function of the feed-forward loop network motif. *Proc. Natl. Acad. Sci. USA* **100**, 11980–11985 (2003).

[27] Villas Boas, P. R., Rodrigues, F. A., Travieso, G. & da Fontoura Costa, L. Chain motifs: The tails and handles of complex networks. *Phys. Rev. E* **77** (2008).

[28] Drossel, B., Mihaljev, T. & Greil, F. Number and Length of Attractors in a Critical Kauffman Model with Connectivity One. *Phys. Rev. Lett.* **94**, 088701 (2005).

[29] Ingram, P. J., Stumpf, M. P. & Stark, J. Network motifs: structure does not determine function. *BMC Genomics* **7**, 108 (2006).

[30] Albert, R. & Othmer, H. G. The topology of the regulatory interactions predicts the expression pattern of the segment polarity genes in Drosophila melanogaster. *J. Theor. Biol.* **223**, 1–18 (2003).

[31] Hooper, J. E. & Scott, M. P. The molecular genetic basis of positional information in insect segments. In *Early Embryonic Development of Animals*, 1–48 (Springer, 1992).

[32] von Dassow, G., Meir, E., Munro, E. M. & Odell, G. M. The segment polarity network is a robust developmental module. *Nature* **406**, 188–192 (2000).

[33] Willadsen, K. & Wiles, J. Robustness and state-space structure of Boolean gene regulatory models. *J. Theor. Biol.* **249**, 749–765 (2007).

[34] Li, F., Long, T., Lu, Y., Ouyang, Q. & Tang, C. The yeast cell-cycle network is robustly designed. *Proc. Natl. Acad. Sci. USA* **101**, 4781–4786 (2004).

[35] Espinosa-Soto, C., Padilla-Longoria, P. & Alvarez-Buylla, E. R. A gene regulatory network model for cell-fate determination during arabidopsis thaliana flower development that is robust and recovers experimental gene expression profiles. *The Plant Cell Online* **16**, 2923–2939 (2004).

[36] Chaos, Á. *et al.* From Genes to Flower Patterns and Evolution: Dynamic Models of Gene Regulatory Networks. *Journal of Plant Growth Regulation* **25**, 278–289 (2006).

[37] Kauffman, S., Peterson, C., Samuelsson, B. & Troein, C. Genetic networks with canalyzing Boolean rules are always stable. *Proc. Natl. Acad. Sci. USA* **101**, 17102–17107 (2004).

[38] Reichhardt, C. J. O. & Bassler, K. Canalization and symmetry in boolean models for genetic regulatory networks. *Physica A* **40**, 4339–4350 (2007).

[39] Marques-Pita, M., Manicka, S., Teuscher, C. & Rocha, L. M. Effective Connectivity as an Order Parameter in Random Boolean Networks Submitted (2018).

[40] Moreira, A. A. & Amaral, L. A. N. Canalizing kauffman networks: Nonergodicity and its effect on their critical behavior. *Phys. Rev. Lett.* **94**, 218702 (2005).

[41] Conrad, M. The geometry of evolution. *BioSystems* **24**, 61–81 (1990).
